# Genome-wide patterns of homozygosity provide clues about the population history and adaptation of goats

**DOI:** 10.1186/s12711-018-0424-8

**Published:** 2018-11-19

**Authors:** Francesca Bertolini, Tainã Figueiredo Cardoso, Gabriele Marras, Ezequiel L. Nicolazzi, Max F. Rothschild, Marcel Amills, the AdaptMap consortium

**Affiliations:** 1 Department of Animal Science, Iowa State University, Ames, IA 50011 USA; 2 National Institute of Aquatic Resources, Technical University of Denmark (DTU), Lyngby, 2800 Denmark; 3 Centre for Research in Agricultural Genomics (CRAG), CSIC-IRTA-UAB-UB, Campus, Universitat Autonoma de Barcelona, 08193 Bellaterra, Barcelona Spain; 4 Fondazione Parco Tecnologico Padano (PTP), 26900 Lodi, Italy

## Abstract

**Background:**

Patterns of homozygosity can be influenced by several factors, such as demography, recombination, and selection. Using the goat SNP50 BeadChip, we genotyped 3171 goats belonging to 117 populations with a worldwide distribution. Our objectives were to characterize the number and length of runs of homozygosity (ROH) and to detect ROH hotspots in order to gain new insights into the consequences of neutral and selection processes on the genome-wide homozygosity patterns of goats.

**Results:**

The proportion of the goat genome covered by ROH is, in general, less than 15% with an inverse relationship between ROH length and frequency i.e. short ROH (< 3 Mb) are the most frequent ones. Our data also indicate that ~ 60% of the breeds display low *F*_ROH_ coefficients (< 0.10), while ~ 30 and ~ 10% of the goat populations show moderate (0.10 < *F*_ROH_ < 0.20) or high (> 0.20) *F*_ROH_ values. For populations from Asia, the average number of ROH is smaller and their coverage is lower in goats from the Near East than in goats from Central Asia, which is consistent with the role of the Fertile Crescent as the primary centre of goat domestication. We also observed that local breeds with small population sizes tend to have a larger fraction of the genome covered by ROH compared to breeds with tens or hundreds of thousands of individuals. Five regions on three goat chromosomes i.e. 11, 12 and 18, contain ROH hotspots that overlap with signatures of selection.

**Conclusions:**

Patterns of homozygosity (average number of ROH of 77 and genome coverage of 248 Mb; *F*_ROH_ < 0.15) are similar in goats from different geographic areas. The increased homozygosity in local breeds is the consequence of their small population size and geographic isolation as well as of founder effects and recent inbreeding. The existence of three ROH hotspots that co-localize with signatures of selection demonstrates that selection has also played an important role in increasing the homozygosity of specific regions in the goat genome. Finally, most of the goat breeds analysed in this work display low levels of homozygosity, which is favourable for their genetic management and viability.

**Electronic supplementary material:**

The online version of this article (10.1186/s12711-018-0424-8) contains supplementary material, which is available to authorized users.

## Background

Runs of homozygosity (ROH) can be defined as genomic regions that display a series of consecutive homozygous genotypes [1]. Their length and frequency depend on a complex array of factors including demography, recombination, and selection [2]. There is convincing evidence that demographic history has had a key influence on the genomic patterns of homozygosity in several domestic animal species [3]. While long ROH reflect recent inbreeding, which can be caused by population decline, unbalanced paternal contributions and selection, short and abundant ROH are often due to ancestral family relatedness [4]. Local recombination rate is negatively correlated with ROH frequency because recombination events decrease the probability that an individual possesses two copies of the same long haplotype [5]. In pigs, the largest ROH are more frequent in regions of low recombination and ROH distribution is negatively correlated with GC content [6]. Regions of low recombination were also detected across the sheep genome [7]. Selection is another important evolutionary force that can increase homozygosity. Positive selection to improve productive/reproductive traits and maintain breed standards can also decrease variability in targeted regions of the genome, and therefore ROH might result from footprints of selection (signatures of selection) [8, 9].

The recent availability of a caprine high-throughput genotyping chip [10] and a reference goat genome [11, 12] has made it possible to characterize the genomic patterns of homozygosity of several populations from Egypt [13], Spain and Africa [14], Switzerland [15] and Italy [16]. Moreover, combining information provided by the genomic distribution of ROH and selection statistics (e.g. *F*_ST_, iHS and hapFLK) has facilitated the identification of several genomic regions under positive selection in goats [13, 15, 17]. However, a comprehensive picture of the genome-wide patterns of homozygosity in goats sampled at a worldwide scale is still lacking. By comparing a wide range of caprine populations that differ in geographic origin, inbreeding and admixture levels and that undergo different management and selection pressures, we investigated the impact of such factors on the abundance and distribution of ROH in the goat genome.

## Methods

### Sampling and data filtering

The AdaptMap dataset was initially composed of samples collected from 4653 goats from 130 breeds and 14 crossbred populations that were genotyped with the Goat SNP50 BeadChip; SNP genomic coordinates were based on the ARS1 reference genome [11]. Animal and SNP quality filtering were performed with the PLINK software [18, 19] and in-house scripts by applying the following criteria of exclusion: (1) individual genotype call rate lower than 0.96; (2) SNP call rate lower than 0.98; (3) minor allele frequency = 0 i.e. no monomorphic markers in the whole dataset; and (4) unmapped SNPs or SNPs on sex chromosomes.

Highly related individuals (pairwise identity-by-state higher than 0.99) were also removed from the dataset. Moreover, in populations with more than 50 individuals, a random sampling selection procedure implemented in the BITE R package [20] was used to retrieve representative samples of 50 individuals for use in further analyses. For additional details, see [21]. In all analyses, only populations with more than 10 animals were considered, except for the comparison of ROH patterns in purebred versus admixed populations, for which all crossbred populations were taken into consideration regardless of their sample size. After these filtering steps, the final dataset included 3171 animals belonging to 105 breeds and 12 crossbred populations (see Additional file 1: Table S1) and 46,654 SNPs.

To investigate the factors that influence the patterns of homozygosity in the goat genome, we performed comparisons based on (1) population characteristics, (2) geographical origin and (3) sampling locations of transboundary breeds.

### Comparison (1) based on population characteristics

This comparison was based on three population characteristics: (1) large versus small size populations, where breeds with a small population size include a few hundreds or thousands of individuals and breeds with a large population size have a census of at least 20,000 individuals, although most of them are in the range of hundreds of thousands of individuals or even millions; (2) traditional versus improved breeds: improved breeds are those that have undergone intensive programs of selection for milk i.e. Maltese, Murciana, Toggenburg or Saanen or meat (e.g. Boer); and (3) crossbreds versus purebred breeds, when available in the dataset.

### Comparison (2) based on geographical origin

Goats were sampled from: (1) America (South America, no subgroups); (2) Oceania (no subgroups); (3) Asia (Central Asia and Near East subgroups); (4) Europe (Central Europe, North Europe, and South Europe subgroups) and (5) Africa (Central West Africa, East Africa, North Africa and South Africa subgroups).

### Comparison (3) based on sampling locations of transboundary breeds

Transboundary breeds collected from multiple locations (Alpine, Boer, Angora, Nubian, and Saanen) were split into subpopulations according to the geographic area where they were sampled (all subgroups were represented by at least 10 animals).

### Data analyses

We used the Zanardi software [22] for ROH analysis of each individual with the following parameters: ROH_SNP (minimum number of SNPs to call a ROH) = 15; ROH_MAXMIS (maximum number of missing SNP per ROH) = 1; ROH_MAXHET (maximum number of heterozygous SNP per ROH) = 1 and ROH_MINLEN (minimum length—in Mb—of ROH) = 1. For each breed, the average fraction of the genome that contains ROH was calculated (*F*_ROH_) by considering the total length (2.92 Gb) of the most recent caprine assembly version ARS1 [11]. For each animal, we calculated the number of detected ROH and ROH coverage. Then, for Comparisons (1) and (2), we used a generalized least squares model implemented in the *nlme* package (R software v.2.15.3) by assuming inequality of the variances associated with each one of the two parameters outlined above (ROH number and coverage) and each group:

$$Y_{i} = {\mathbf{X}}_{i} {\varvec{\upbeta}} + \varepsilon_{i} ,$$

$$\varepsilon_{i } \sim N \left( { 0, \sigma^{2 } {\varvec{\Lambda}}_{i} } \right),$$

$${\text{and}}\quad i = 1, \ldots ,m,$$

where ${\varvec{\upbeta}}$ is a vector of the fixed effect “breed” (*m* levels), ${\mathbf{X}}_{i}$ is an incidence matrix relating $Y_{i }$ to ${\varvec{\upbeta}}$, and ${\varvec{\Lambda}}_{i}$ is a positive-definite matrix of the variances and covariances of the within-group errors. For full details of this methodology, see Pinheiro and Bates [23]. For both analyses of ROH number and genome coverage, the least square means of each subgeographical group were then compared on a pairwise basis with a Wald univariate test of significance [24] and multiple testing was adjusted with the Bonferroni correction.

In Comparison (2), ROH were classified into seven length classes (0–3 Mb, 3–5 Mb, 5–10 Mb, 10–15 Mb, 15–20 Mb, 20–25 Mb, and > 30 Mb). For each subgroup and length class, ROH were summed and averaged according to the number of animals included in each subgroup. The –save option was used to retain the output derived from the analyses that provided, for each SNP, the percentage of animals that have a ROH in a given position (*H* score). This information was used to detect ROH hotspots across the goat genome by considering regions that contained at least three SNPs above the top 0.998 of the overall SNP distribution. The *H* score that represents this distribution varied between comparisons. Then, we performed a gene search within the common ROH hotspots by using the most recent available annotated genome version, ARS1 [11].

For transboundary breeds that were raised in multiple countries, the summary statistics of the percentage of animals that have a ROH in a given position were calculated for each country and breed and then standardized to compare the locus-specific divergence for each location based on *H* score:

$$SHD_{i} = \mathop \sum \limits_{i \ne j} \frac{{HD^{ij} - E\left( {HD^{ij} } \right)}}{{sd\left( {HD^{ij} } \right)}},$$

where *HD*^*ij*^ is the difference in *H* scores between two subpopulations *i* and *j*, and *E*(*HD*^*ij*^) and *sd*(*HD*^*ij*^) denote the expected value and standard deviation of *HD* between the *i*th and *j*th sub-populations. These analyses were performed by using the R computing environment (https://www.r-project.org/) and implementing the approach suggested by Akey et al. [25] and modified by Bertolini et al. [26]. To provide a detailed view of the divergence between each country classification, we carried out the comparisons by contrasting the standard deviation of a subpopulation sampled in a given country versus all the subpopulations raised in the remaining countries where the transboundary breed was sampled. For the Nubian transboundary breed, which in our dataset was exclusively distributed in Egypt and Argentina, the comparison was made on a pairwise basis. Genomic regions that were represented by at least three consecutive SNPs and displayed the largest differences in *H* scores (SHD > 5) were considered in these analyses.

## Results

### Calculation of *F*_ROH_ values in goat breeds

The average fraction of the genome that contains ROH in each analyzed breed is provided in Additional file 2: Figure S1 and Additional file 1: Table S2. These data show that ~ 60% of the breeds display low *F*_ROH_ coefficients (< 0.10), while ~ 30 and ~ 10% of the populations show moderate (0.10 < *F*_ROH_ < 0.20) or high (> 0.20) *F*_ROH_ values. A high variability in the magnitude of *F*_ROH_ coefficients within breeds was also observed in our dataset. It is interesting to note that several of the caprine populations with the highest *F*_ROH_ values are raised on islands and have undergone prolonged geographic isolation (see Additional file 2: Figure S1 and Additional file 1: Table S2), and this is further discussed in an accompanying paper [27]. Other goat breeds with high *F*_ROH_ coefficients were four breeds from Pakistan: Kachan (KAC), Kamori (KAM), Bari (BRI) and Barbari (BAB) (*F*_ROH_ = 0.20–0.25), and Boer (BOE), one of the most improved goat breeds (*F*_ROH_ = 0.21).

### Patterns of homozygosity in goat breeds with a broad geographic distribution

**Fig. 1:**
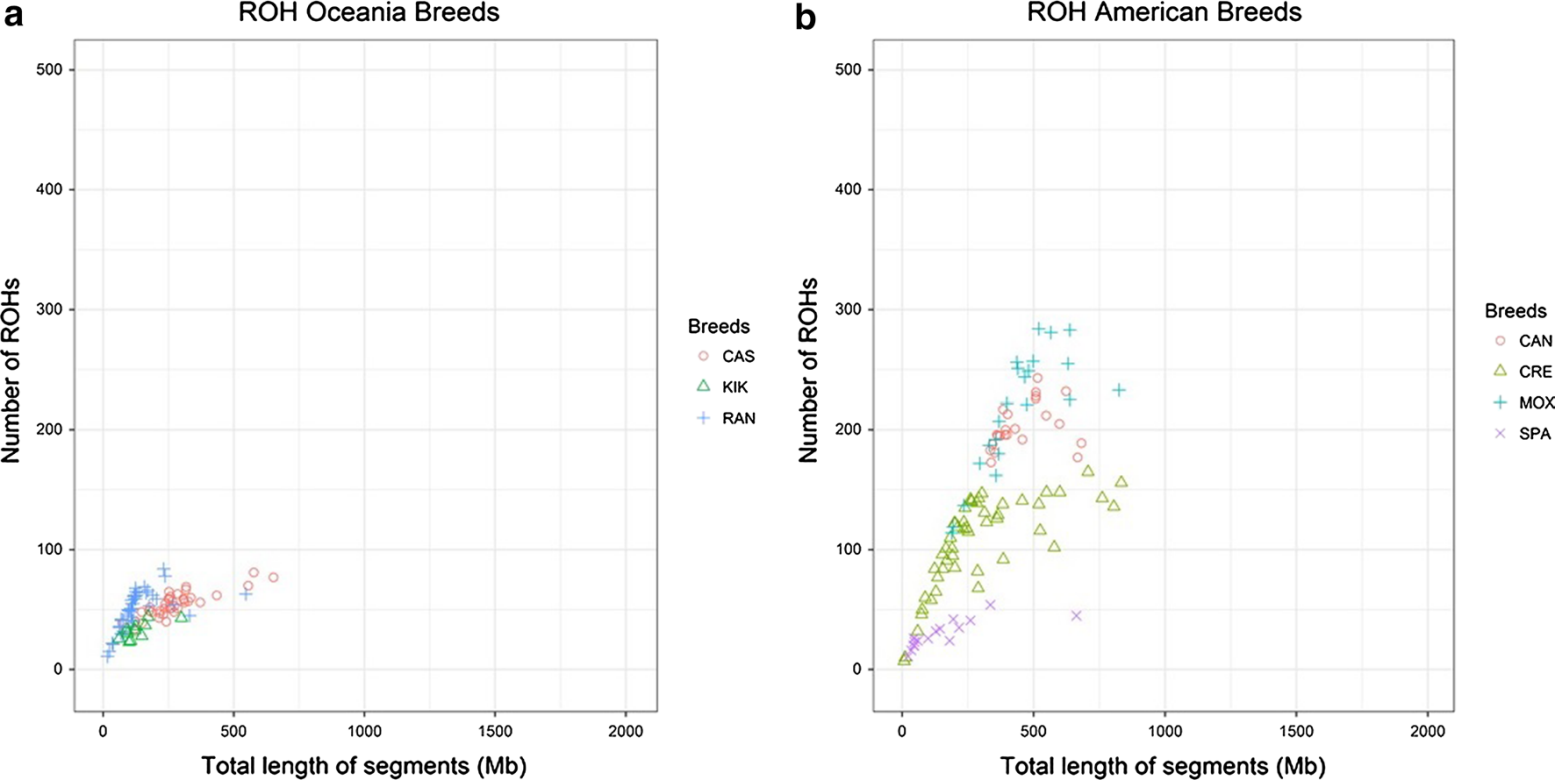
Genomic patterns of homozygosity in goats from Oceania (**a**) and America (**b**). The total length of the genome covered by ROH and the total number of ROH are plotted on the x- and y-axis, respectively. Breed acronyms: **a** CAS: Cashmere; KIK: Kiko; RAN: Rangeland and **b** CAN: Caninde; CRE: Creole; MOX: Moxoto; SPA: Spanish

**Fig. 2:**
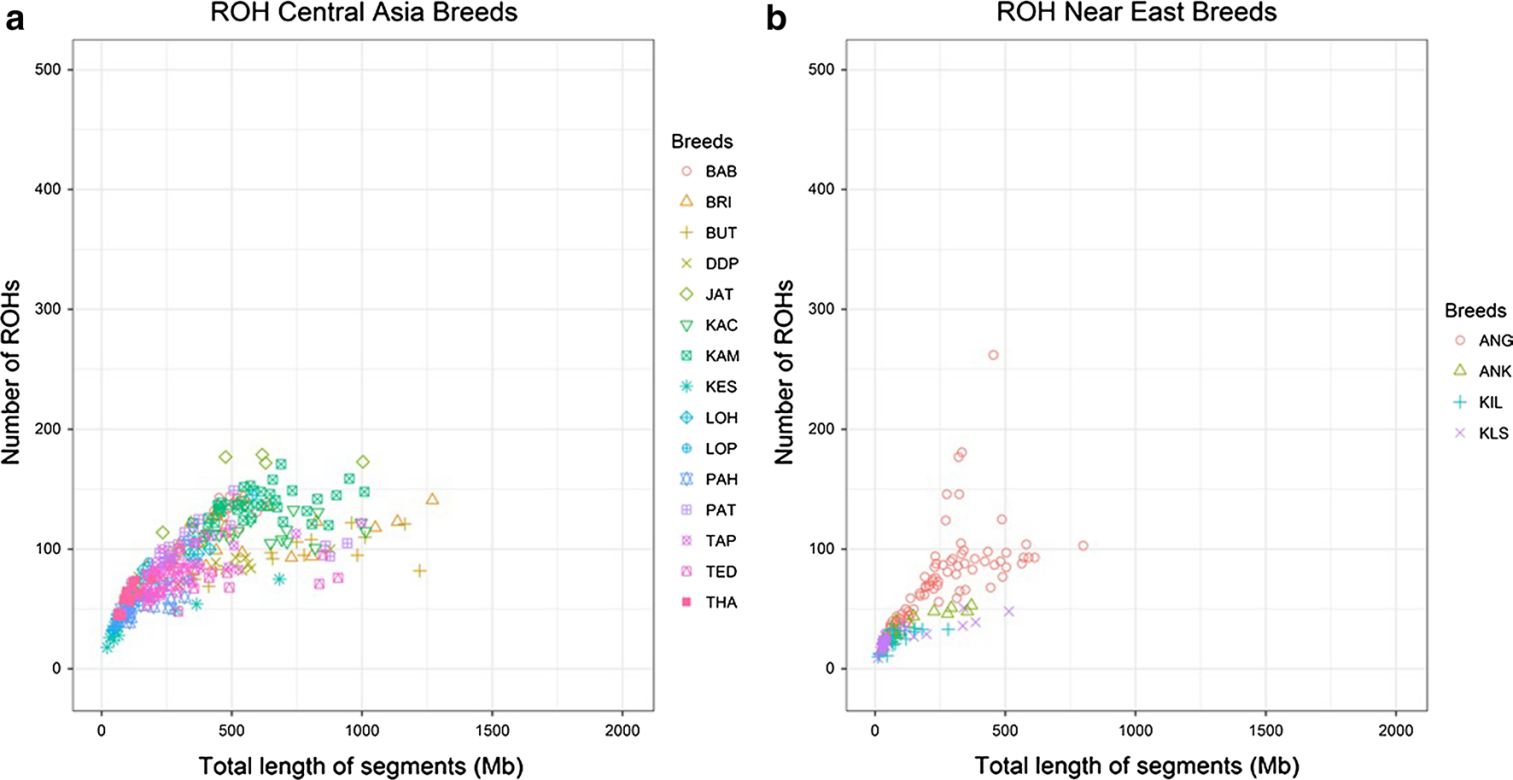
Genomic patterns of homozygosity in goats from Asia: **a** Central Asia and **b** Near East. The total length of the genome covered by ROH and the total number of ROH are plotted on the x- and y-axis, respectively. Breed acronyms: **a** BAB: Barbari; BRI: Bari; BUT: Bugituri; DDP: Dera Din Panah; JAT: Jattan; KAC: Kachan; KAM: Kamori; KES: Koh-e-sulmani; LOH: Lohri; LOP: Local_Pothohari; PAH: Pahari; PAT: Pateri; TAP: Tapri; TED: Teddi; THA: Thari and **b** ANG: Angora; ANK: Ankara; KIL: Kil; KLS: Kilis

**Fig. 3:**
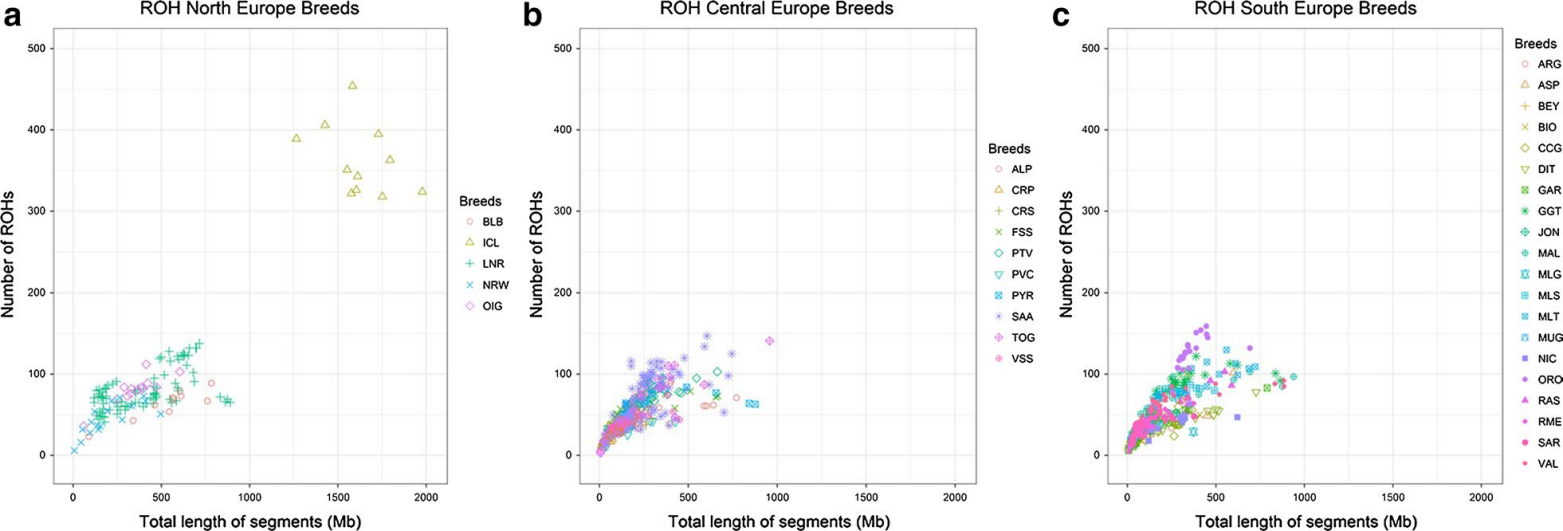
Genomic patterns of homozygosity in goats from Europe: **a** North Europe, **b** Central Europe and **c** South Europe. The total length of the genome covered by ROH and the total number of ROH are plotted on the x- and y-axis, respectively. Breed acronyms: **a** BLB: Bilberry; ICL: Icelandic; LNR: Landrace_goat; NRW: Norwegian; OIG: Old_Irish_goat; **b** ALP: Alpine; CRP: Carpathian; CRS: Corse; FSS: Fosses; PTV: Poitevine; PVC: Provençale; PYR: Pyrenean; SAA: Saanen; TOG: Toggenburg; VSS: Valpassiria and **c** ARG: Argentata; ASP: Aspromontana; BEY: Bermeya; BIO: Bionda_dell’Adamello; CCG: Ciociara_Grigia; DIT: Di_Teramo; GAR: Garganica; GGT: Girgentana; JON: Jonica; MAL: Mallorquina; MLG: Malagueña; MLS: Maltese_Sarda; MLT: Maltese; MUG: Murciano-Granadina; NIC: Nicastrese; ORO: Orobica; RAS: Blanca_de_Rasquera; RME: Rossa_Mediterranea; SAR: Sarda; VAL: Valdostana

**Fig. 4:**
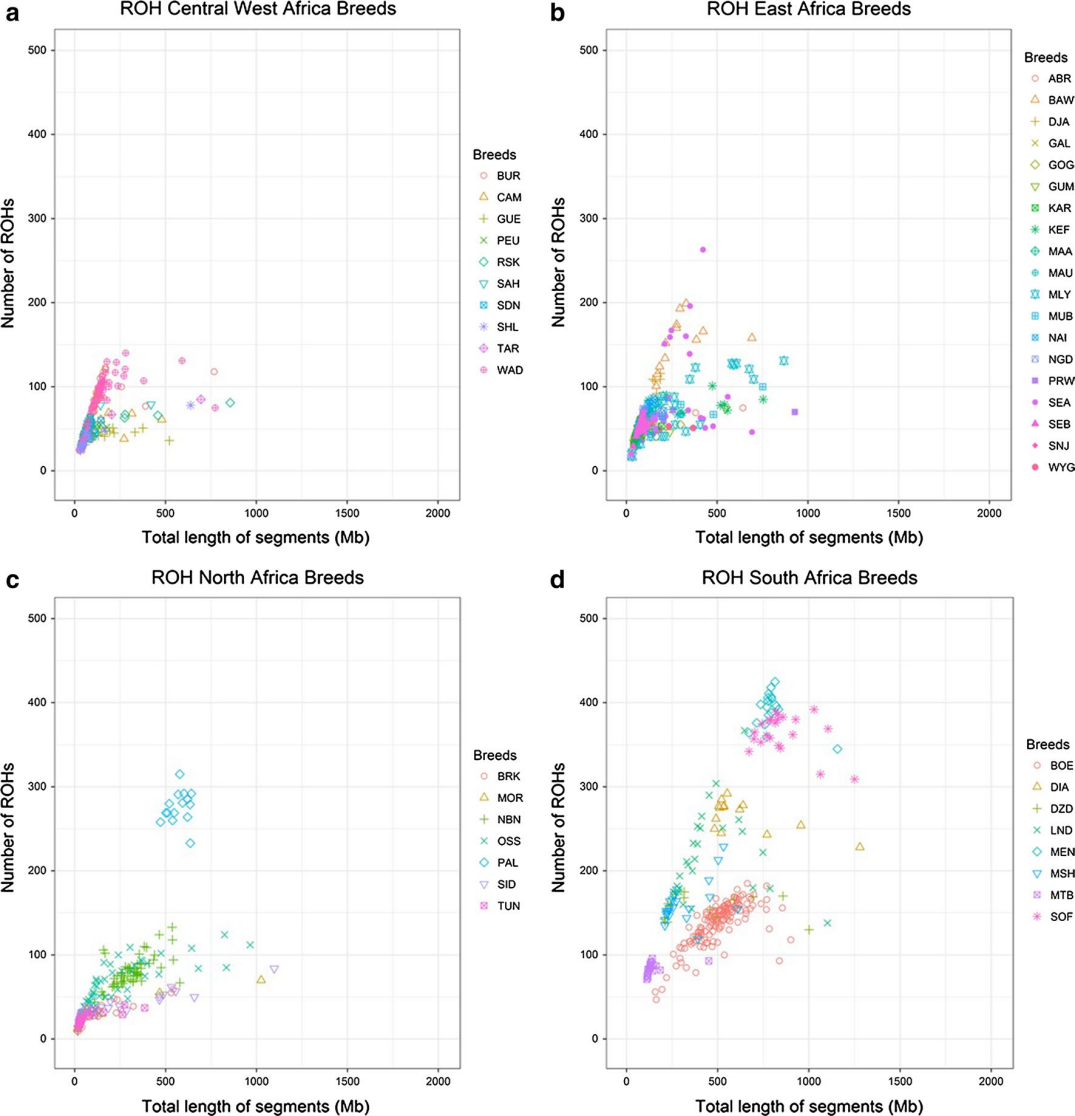
Genomic patterns of homozygosity in goats from Africa: **a** Central West Africa, **b** East Africa, **c** North Africa and **d** South Africa. The total length of the genome covered by ROH and the total number of ROH are plotted on the x- and y-axis, respectively. Breed acronyms: **a** BUR: Burundi_goat; CAM: Cameroon_goat; GUE: Guera; PEU: Peulh; RSK: Red_Sokoto; SAH: Sahel; SDN: Soudanaise; SHL: Sahel; TAR: Targui; WAD: West_African_goat; **b** ABR: Abergelle; BAW: Balaka-Ulongwe; DJA: Djallonke; GAL: Galla; GOG: Gogo; GUM: Gumez; KAR: Karamonja; KEF: Keffa; MAA: Maasai; MAU: Maure; MLY: Malya; MUB: Mubende; NAI: Naine; NGD: Nganda; PRW: Pare_White; SEA: Small_East_Africa; SEB: Sebei; SNJ: Sonjo; WYG: Woyito_Guji; **c** BRK: Barki; MOR: Moroccan_goat; NBN: Nubian; OSS: Oasis; PAL: Palmera; SID: Saidi; TUN: Tunisian and **d** BOE: Boer; DIA: Diana; DZD: Dedza; LND: Landin; MEN: Menabe; MSH: Mashona; MTB: Matebele; SOF: Sofia

Figures 1, 2, 3 and 4 and Additional file 1: Table S3, show the number and length of ROH per individual (p.i.) divided by sub-geographical groups (Comparison 2). The average number and length of ROH calculated across all the animals in the comparison were equal to 77 ROH and 248 Mb. Results of the comparison of the least square means between the sub-geographical groups are in Additional file 1: Table S4. All the comparisons showed significant differences (P_adj-value_ < 0.05), for at least one of the two parameters considered (ROH number and coverage), except for Central_Asia-North_Europe, Central_Europe-North_Africa, Central_Europe-Oceania, Central_Europe-South_Europe, Central_Western_Africa-East_Africa, Near_East-North_Africa, North_Africa-South_Europe, North_Africa-Oceania and Oceania-South_Europe. It is interesting to note that the average ROH number and coverage for goats from Oceania (49 ROH and 182.19 Mb) were significantly lower (P_adj-value_ < 0.0001) than those for goats from America (136 ROH and 333 Mb), see Fig. 1 and Additional file 1: Table S3 and Table S4. This group of Oceania goats had also the lowest standard deviation for both ROH regions and ROH coverage among all comparisons (see Additional file 1: Table S3). For goats from Asia (Fig. 2), the average ROH number and coverage were significantly lower in goats from the Near East (P_adj-value_ < 0.0001), with 60 ROH and 210.64 Mb, than from Central Asia (90 ROH and 260.64 Mb). As shown in Figs. 3 and 4, several insular European (e.g. Icelandic) and African (e.g. goats from Madagascar) breeds showed large ROH numbers (> 400) and high ROH coverage (1000–2000 Mb).

**Fig. 5:**
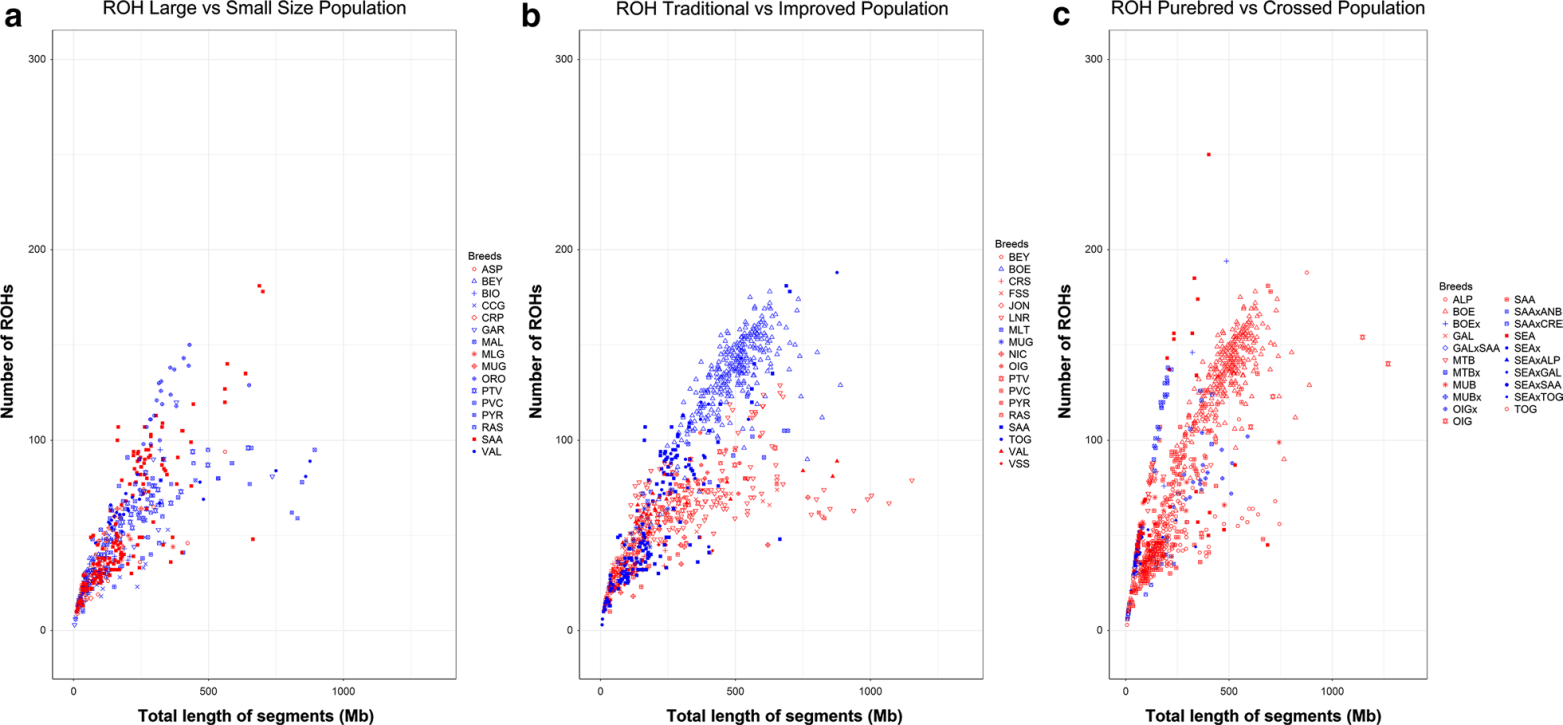
Genomic patterns of homozygosity in a selected set of populations specialized in either dairy or meat traits with **a** small (blue) and large (red) size, **b** subject to a moderate/strong (blue) or mild (red) selection and **c** crossbred (blue) and purebred (red) genetic composition. The total length of the genome covered by ROH and the total number of ROH are plotted on the x- and y-axis, respectively. Breed acronyms: **a** ASP: Aspromontana; BEY: Bermeya; BIO: Bionda_dell’Adamello; CCG: Ciociara_Grigia; CRP: Carpathian; GAR: Garganica; MAL: Mallorquina; MLG: Malagueña; MUG: Murciano-Granadina; ORO: Orobica; PTV: Poitevine; PVC: Provençale; PYR = Pyrenean; RAS: Blanca_de_Rasquera; SAA: Saanen; VAL: Valdostana; **b** BEY: Bermeya; BOE: Boer; CRS: Corse; FSS: Fosses; JON: Jonica; LNR: Landrace_goat; MLT: Maltese; MUG: Murciano-Granadina; NIC: Nicastrese; OIG: Old_Irish_goat; PTV: Poitevine; PVC: Provençale; PYR: Pyrenean; RAS: Blanca_de_Rasquera; SAA: Saanen; TOG: Toggenburg; VAL: Valdostana; VSS: Valpassiria and **c** ALP: Alpine; BOE: Boer; BOEx: Admixed_Boer; GAL: Galla; GALxSAA: GallaxSaanen; MTB: Matebele; MTBx: Matebele_cross; MUB: Mubende; MUBx: Admixed; OIG: Old_Irish_goat; OIGx: Old_Irish_goat_cross; SAA: Saanen; SAA × ANB: Saanen × Anglo_NubianF2; SAA × CRE: Saanen × Creole; SEA: Small_East_Africa; SEAx: Admixed_Small_East_Africa; SEAxALP: Small_East_AfricaxAlpine; SEAxGAL: Small_East_Africa × Galla; SEAxSAA: Small_East_Africa × Saanen; SEAxTOG: Small_East_Africa × Toggenburg; TOG: Toggenburg

To understand the effects of demography, admixture and selection on the patterns of homozygosity, we carried out comparisons based on sets of selected caprine breeds. Results of the comparison between continental breeds that have large population sizes (tens or hundreds of thousands of individuals, Murciano-Granadina, Malagueña, Carpathian, Saanen, etc.) and local breeds with relatively small population sizes are in Fig. 5a and Additional file 1: Table S5. In terms of ROH number, the difference between both groups is very significant with local breeds tending to have a larger fraction of the genome covered by ROH. The comparison of highly selected meat and dairy breeds versus traditional populations, in which selection pressure is much lower, highlights remarkable differences in ROH number and coverage (Fig. 5b) and (see Additional file 1: Table S5), with the highest values found for the improved transboundary Boer, Saanen and Toggenburg breeds. In contrast, goats with the highest ROH coverage (> 750 Mb) belong to traditional breeds such as Valdostana and Landrace (Fig. 5b). This difference between crossbred and purebred populations (Fig. 5c) and (see Additional file 1: Table S5) demonstrates that, as expected, total ROH length and number are significantly smaller (P_adj-value_ < 0.0001) in crossbred populations.

**Fig. 6:**
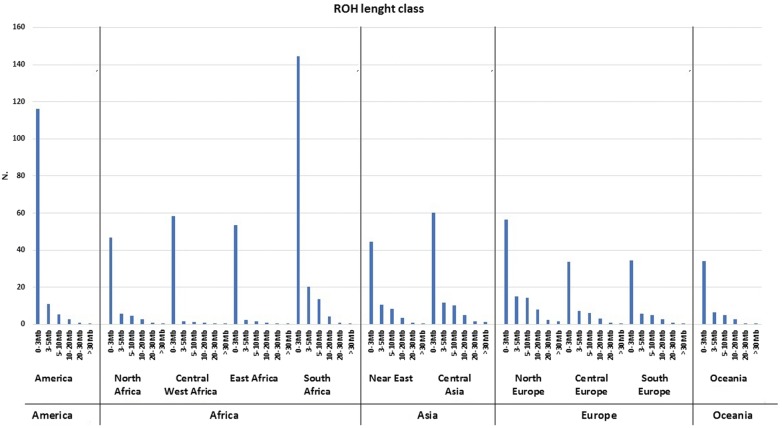
Mean sum of runs of homozygosity per genotyped animal. For each animal, the sum of ROH for each length class (0–3 Mb, 3–5 Mb, 5–10 Mb, 10–20 Mb, 20–30 Mb, > 30 Mb) was calculated and averaged across the different geographical groups

Figure 6 shows the distribution of ROH classes (i.e. classified according to length) across continental groups. Among the six ROH classes under consideration, short ROH (< 3 Mb) are the most frequent ones in all populations, with a wide distribution that spans from an average of 33 ROH p.i. in Central European goats to 144 ROH p.i. in South African goats. The distribution of the 3–10 Mb and 10–30 Mb length classes ranged between 1.4 (Central West Africa) to 20 (South Africa) ROH p.i. and 2.2 (Central West Africa) to 24 (North Europe) ROH p.i., respectively. Finally, the largest ROH class (> 30 Mb) was the rarest one, with frequencies ranging from 0.3 (East and South Africa) to 1.4 (North Europe) ROH p.i.

### Genomic distribution of ROH in the caprine genome and differences between transboundary breeds

Analyses across the continental and subcontinental divisions revealed several partial or complete ROH overlaps (top 0.998 regions) across all populations listed in Additional file 1: Table S4. Several ROH were exclusively present in goats from a single continent. For instance, on *Capra hircus* chromosome (CHI) CHI18, the 1.7-Mb (14.64–16.38 Mb) and 842-kb ROH (26.83–27.67 Mb) were specific to Asian and American breeds, respectively. Moreover, six ROH ranging in size from 107 kb to 1 Mb on CHI3 (110.15–111.16 Mb), CHI5 (95.67–96.54 Mb), CHI7 (59.82–59.92 Mb), CHI8 (43.94–44.62 Mb), CHI11 (94.23–94.50 Mb) and CHI12 (48.30–48.44 Mb) were exclusively detected in goats from Oceania.

**Fig. 7:**
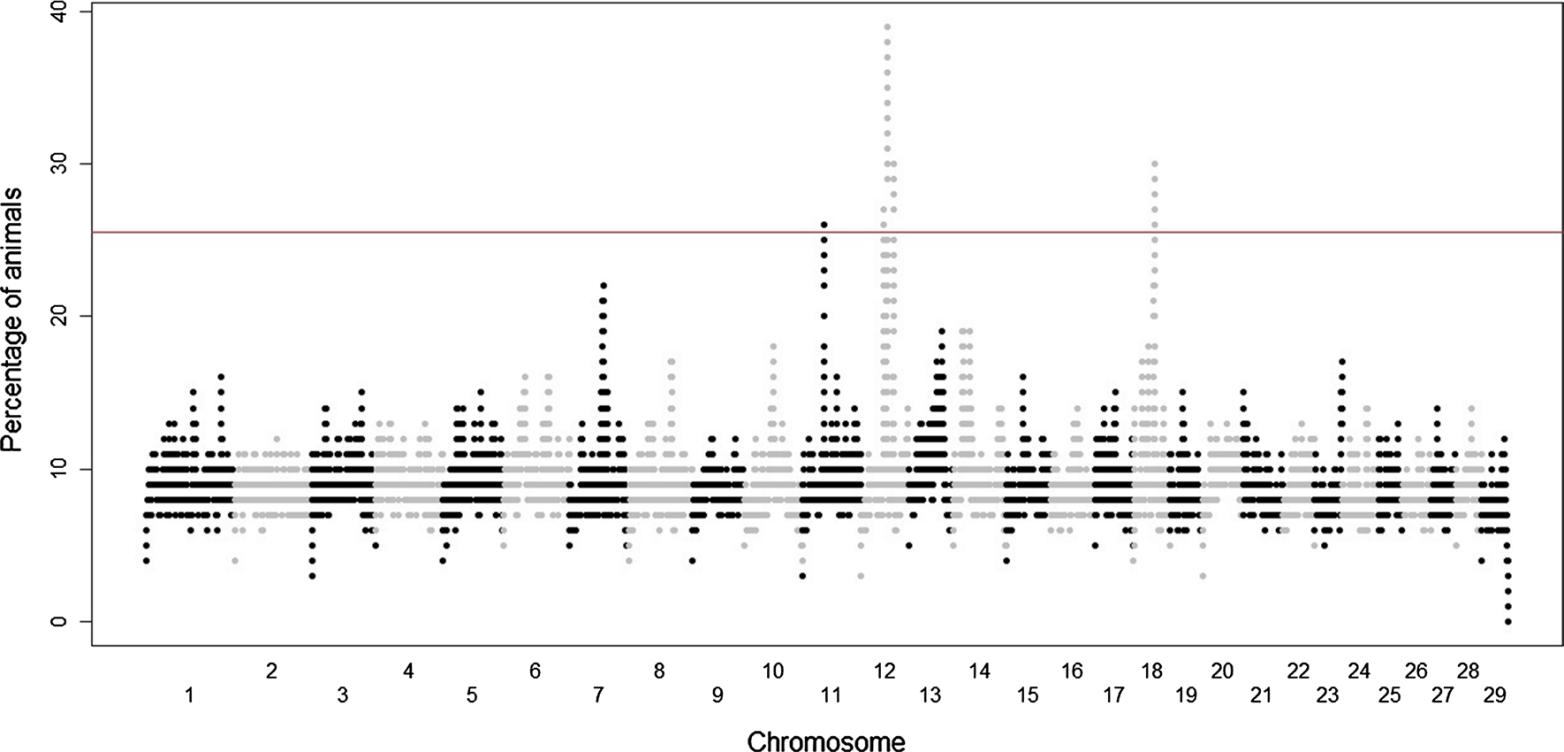
Incidence of each SNP in a run of homozygosity across the worldwide goat dataset. The red line indicates the threshold of 26% corresponding to the 0.998 percentile of the overall SNP distribution

Five regions on three chromosomes, i.e. CHI11, 12 and 18, contained ROH that are present in the highest percentage of animals (Fig. 7). A ROH on CHI11 (37.79–38.33 Mb) was particularly frequent in European and African goats. Three regions on CHI12 were also highly homozygous in a broad array of populations i.e. 43.63–44.53 Mb (Europe), 50.02–51.38 Mb (all continents) and 60.11–61.02 Mb (Europe, Africa, Oceania and Asia). Finally, one ROH on CHI18 (36.22–37.01 Mb) was highly frequent in goats from Europe, Africa, and Asia (see Additional file 1: Table S6). Overall, these regions contained 68 annotated coding genes (see Additional file 1: Table S6), including *gap junction protein beta 6* (*GJB6*), *Sin3A associated protein 18* (*SAP18*), and *gap junction protein beta 2* (*GJB2*).

We compared the patterns of homozygosity in transboundary breeds raised in different locations based on the *H* scores derived from the locus-specific divergence analyses (see Additional file 3; and Additional file 1: Table S7). In general, subpopulations from one transboundary breed did not show marked differences in their homozygosity parameters. However, several genomic regions diverged significantly between subpopulations. In the Alpine breed, sampled in Italy (IT), Switzerland (CH) and France (FR), two regions of about 760 kb on CHI3 (91.54–92.29 Mb) and 13 (62.90–63.69 Mb) were specific to Alpine goats sampled in Switzerland. In contrast, a 1.12-Mb region on CHI11 (94.31–95.44 Mb) was exclusive to Italian Alpine goats. In the Angora breed, one 241.77-kb region on CHI14 (53.14–53.38 Mb) and two longer regions of 2.28 and 1.75 Mb on CHI23 (20.44–22.29 Mb and 15.05–16.80 Mb) differentiated animals sampled in South Africa. In Saanen goats, a 247.22-kb region on CHI6 (29.89–30.14 Mb) was specific to Swiss Saanen goats and a 688.71-kb region on CHI13 (50.40–51.09 Mb) was exclusive to French Saanen goats.

## Discussion

### Effects of population history and geographic distribution on homozygosity patterns

The patterns of homozygosity that were identified in a worldwide sample of goats are similar to those previously reported in 891 cattle from multiple breeds [28] and in 3191 sheep from six commercial populations [29], i.e. the proportion of the genome covered by ROH is, in general, less than 15% and there is an inverse relationship between ROH length and frequency (Fig. 6). In this regard, it is worth mentioning that low-density chips (as that used here) do not detect small ROH accurately [28], which are the most frequent ones in outbred domestic animals [6, 30]. This means that the true levels of homozygosity of the caprine breeds analyzed in the current work may be underestimated.

The levels of homozygosity were remarkably low (Fig. 2) in Near Eastern breeds except for a few Angora individuals, and in the European group. These findings agree well with previous studies that indicated Eastern Anatolia as a primary domestication center for goats, which subsequently dispersed into Europe, where breed formation was probably more systematic than in western Asia [21, 31]. In humans, a worldwide analysis of ROH patterns revealed a positive correlation between proportion of short and intermediate ROH and distance to Africa, the birthplace of humankind [5]. These results are consistent with the idea that, in populations undergoing serial founder effects (during successive range expansions), homozygosity tends to increase. In Pakistan, four breeds (Bari, Barbari, Kamori, and Kachan) displayed *F*_ROH_ values higher than 0.20 (see Additional file 1: Table S2) and large ROH numbers, which might be explained by an ancient founder effect associated with the initial dispersal of goats from their domestication center in the Fertile Crescent [32]. An alternative, but not mutually exclusive, explanation would be the occurrence of random consanguineous matings due to an open village breeding system, as reported in certain African bovine breeds [28]. The ROH pattern detected in Oceania populations was similar to that detected in several European and African groups, confirming the findings reported by Colli et al. [21] that goats from Oceania possess African or African × European genetic backgrounds.

The patterns of homozygosity in goats from Oceania and America are quite different although these two populations were founded around 200 and 500 years ago, respectively. According to our data, ROH number and length are much smaller in goats sampled in Oceania than in those sampled in America. In principle, a recent founder effect should have resulted in a larger number of ROH, as observed for American goats (many individuals with more than 100 ROH) but not for Oceanian goats. The most likely explanation for these unexpected results is the extensive crossbreeding of goats from Oceania. For instance, Rangeland goats are composed of a mixture of Angora, Cashmere, Anglo-Nubian, British Alpine, Saanen and Toggenburg breeds [33], and Kiko is a synthetic breed recently created by crossing New Zealand feral goats with multiple improved exotic breeds (https://www.jumpingfrogfarm.com/history-of-kikos). As we will explain in the next section, population admixture and crossbreeding both contribute to the disruption of long homozygous stretches and decrease in global autozygosity levels.

Regarding the transboundary breeds, in general we detected no major differences across subpopulations through locus-specific divergence analyses, which is probably mainly due to the recent worldwide dispersal of these breeds because of the intensification of goat production, artificial insemination and the existence of an efficient transportation network across the globe. Hardiness and robustness of goats also facilitate the shipment of improved breeders to distant countries. However, ROH regions of high divergence were identified in several comparisons. For example, Alpine goats that were sampled from a limited and close geographical area (Italy, France, and Switzerland) showed different ROH distributions particularly on CHI3 (91.54–92.29 Mb) and 13 (62.90–63.69 Mb) for the subpopulation sampled in Switzerland, and on CHI11 (94.31–95.44 Mb) for the subpopulation sampled in Italy. Differences in the genome-wide diversity patterns of the Alpine goats sampled in these countries were also observed in the admixture analyses that were carried out by Colli et al. [21] and covered the whole AdaptMap dataset. These admixture analyses revealed at K = 50 a clear genetic differentiation between Alpine goats sampled in Switzerland versus those sampled in Italy and France. In the Angora breed, regions of high divergence were detected on CHI14 (53.14–53.38 Mb) and 23 (20.44–22.29 Mb and 15.05–16.80 Mb) for animals sampled in South Africa. This result is consistent with the genome-wide analysis of diversity mentioned before [21], where the admixture analyses at K = 50 revealed the existence of genetic differences between Angora goats from South Africa and those sampled in other countries. For the Boer breed, no major regions of divergence were detected in our analyses, which is concordant with the admixture analyses performed by Colli et al. [21] who observed genetic differences between Boer subpopulations (e.g., Australia vs. Switzerland) but they tend to be more tenuous than those observed in other transboundary breeds.

### Consequences of population admixture and inbreeding on homozygosity levels

The effects of population admixture on homozygosity patterns are illustrated in Fig. 5b. It is evident that total ROH length is much shorter in crossbred goats than in their purebred counterparts. Iberian cattle, that have been significantly introgressed with African breeds, also show a lower ROH abundance than British breeds that have a single European ancestry [29]. Moreover, a direct relationship between admixture and ROH length has been documented in African cattle populations, such as Kuri and Sheko, which were generated by crossing *Bos taurus* × *Bos indicus* [28]. Szpiech et al. [2] reported that, in humans, long ROH are enriched in variants with a predicted damaging effect. Similarly, in cattle, ROH are more enriched in predicted deleterious variants than non-deleterious variants [34], but, in contrast with humans, this enrichment is more significant for short and medium ROH.

Demography is another important factor that shapes the genomic patterns of homozygosity [6]. Our results indicate that insular goat populations, such as those raised in Iceland or Madagascar, display increased levels of homozygosity, which is discussed in detail in a companion paper [27] and thus not further developed here. It is worth noting that most of the continental populations with a ROH coverage higher than 750 Mb correspond to local breeds such as Valdostana, Pyrenean and Mallorquina (Fig. 5a). One common feature of these breeds is that they have suffered sharp population declines due to competition with more productive transboundary breeds and the progressive abandonment of low-income farming activities. For instance, during the second half of the twentieth century, the Pyrenean breed almost disappeared, while currently it comprises 2800 individuals (http://www.capgenes.com/IMG/pdf_ Pyreneenne_anglais.pdf). With 640 registered individuals, the Valdostana breed is at risk [16], and the Mallorquina breed, with only 16 bucks and 141 does, is critically endangered (http://www.mapama.gob.es). Population reduction often involves a global increase in the levels of inbreeding and autozygosity. For instance, Williams et al. [35] analyzed the genetic diversity of a herd of Chillingham cattle, which has been maintained reproductively closed for 350 years, and found that 90% of the SNPs on the 770K chip were monomorphic. Although autozygosity is often associated with inbreeding depression in domestic animals [3], no decrease in fertility or viability of this cattle herd was observed, probably because artificial selection that was exerted during three centuries has purged damaging alleles [35].

### Several ROH hotspots map to putative selective sweeps

Artificial selection generally results in an increase of the ROH frequency and coverage in the genomes of livestock species (e.g. [36]). The comparison between improved and traditional breeds (Fig. 5c) showed that ROH numbers are larger in highly selected breeds such as Saanen, Toggenburg and Boer. The case of the Boer breed is interesting because its production performance in terms of growth is excellent and, thus, it is used for meat production. Since 1970, this breed is incorporated into the National Mutton Sheep and Goat Performance Testing Scheme [37], which makes Boer one of the first goat breeds routinely involved in a performance test for meat production. Saanen and Toggenburg goats, which have been subjected to artificial selection to improve dairy performance, are well known for their high milk yields. In contrast, local goat breeds, such as Valdostana, display longer ROH probably as a consequence of demographic decline and recent inbreeding.

One of the goals of our study was to detect regions of the genome where ROH were more abundant and to investigate if they coincided with the signatures of selection reported by Bertolini et al. [38]. It should be noted that the ROH analyses do not account for population stratification. Thus, some of the signals may correspond to differentiation between groups of breeds as reported in sheep [7]. However, the vast majority of the genomic regions detected across all comparisons mapped to CHI11, 12 and 18 (see Additional file 1: Table S4). The homozygous region on CHI12 (60–61 Mb) overlaps with a signature of selection that was detected with FLK/hapFLK statistics in several geographical subgroups [38]. In our analyses, this ROH is shared by almost all the continental and sub-continental groups excluding America, Oceania and Central Europe. Indeed, American and Oceanian breeds showed some distinctive and unique hotspot regions.

The ROH on CHI11 (37–38 Mb) is shared by goats from three continents (Europe, Americas and Asia) and, interestingly, a signature of selection related to milk production has been detected in the same genomic region in caprine populations from America, East Africa and Central Europe [38]. The other two ROH hotspots on CHI18 (36–37 Mb) that are shared by goats from Europe, Africa and Asia, were reported as possible signatures of selection for fiber production, but the number of SNPs that support these signatures is small [38]. Two other ROH regions on CHI12 (43–44 Mb and 50–51 Mb) overlap or are close to signatures of selection reported in Barki goats [13] on CHI12 at ~ 49–52 Mb and 44–46 Mb (regions updated on the ARS1 genome version). It is interesting to note that these putative signatures of selection were also reported in Barki sheep [13]. These regions contain genes that are related to ectodermal, nervous system and hearing functions, such as *GJB6* and *GJB2* [39, 40] and gonad development such as *SAP18* [41].

## Conclusions

Patterns of homozygosity can be similar in populations from different geographic areas. Moreover, reduced population size, strong founder effects and geographic isolation are associated with increased levels of homozygosity in goats, while population admixture has the opposite effect. The existence of three ROH hotspots that co-localize with signatures of selection demonstrates that selection has also played an important role in increasing the homozygosity of specific regions of the goat genome. Our results will be useful to define future strategies that aim at ensuring the genetic management of goat resources with a broad geographic distribution and a remarkable impact on the economy of developing countries.

## Additional files

**Additional file 1: Table S1.** Animals used for the analyses. Breed symbol, name and number (N). The country in which samples were collected (Country), the continental and subcontinental groups used for the analyses are reported. **Table S2.** Average fraction of the genome that contains ROH in each one of the breeds under analysis. Breed code (Breed) and average fraction of the genome that contains ROH (*F*_ROH_). Breeds are reported based on *F*_ROH_ increasing values. **Table S3.** Summary statistics of number of ROH regions and genome coverage considering the sub-geographical and continental classification. min: minimum number of ROH regions or coverage detected; max: minimum number of ROH regions or coverage detected; mean: average number of ROH regions or coverage detected; sd: standard deviation from the mean value. **Table S4.** Comparison of pairwise least square means of the sub-geographical comparisons. Comparison: pairwise comparison considered; estimate: estimated difference in LSM; p.value: adjusted Bonferroni *P* value. **Table S5.** Summary statistics of number of ROH regions and genome coverage for comparison 1 and pairwise least square means comparison. min: minimum number of ROH regions or coverage detected; max: maximum number of ROH regions or coverage detected; mean: average number of ROH regions or coverage detected; sd: standard deviation from the mean value; estimate: estimated difference in LSM; p.value: adjusted Bonferroni P-value. **Table S6.** Chromosomal regions with a high level of homozygosity (the top 0.998 percentile of at least three consecutive SNPs) and overlaps (partial or complete) across continents and continental sub-divisions. NP = not present; “-“: no overlap detected; for the regions shared by most of the subgroups (All), the symbol of the genes detected within those regions are reported. See the bold number in the “overlap with other continental/sub-continental” column. (1) *MIR217*; *MIR216B*; *CFAP36*; *PNPT1*; *PPP4R3B; EFEMP1.* (2) *GJB6*; *SAP18*; *MRPL57*; *ATP12A*; *CENPJ*; *MPHOSPH8*; *ZMYM5*; *GJA3*; *GJB2*; *CRYL1*; *IL17D*; *EEF1AKMT1*; *LATS2*; *SKA3*; *ZDHHC20*; *FGF9*; *RNF17*; *PSPC1*; *ZMYM2*; *IFT88*; *XPO4*; *MICU2*; *PARP4.* (3) *MAB21L1*; *DCLK1*; *NBEA.* (4) *TPPP3*; *AGRP*; *CARMIL2*; *PARD6A*; *ENKD1*; *C18H16orf86*; *TSNAXIP1*; *THAP11*; *NUTF2*; *EDC4*; *NRN1L*; *LCAT*; *DPEP3*; *DPEP2*; *DDX28*; *SLC7A6OS*; *LRRC3*6; *ZDHHC1*; *ATP6V0D1*; *FAM65A*; *ACD*; *GFOD2*; *CENPT*; *PSKH1*; *PSMB10*; *DUS2*; *ESRP2*; *PLA2G15*; *SLC7A6*; *SMPD3*; HSD11B2; *CTCF*; *RANBP10*; *NFATC3*; *PRMT7*; *SLC12A4.* **Table S7.** Chromosomal regions displaying a high divergence in homozygosity across countries for the same breeds. For each breed-based analysis, chromosome (chr), start and end of the regions with H ≥ 5 are reported.

**Additional file 2: Figure S1.** *F*_ROH_ calculated in the single breeds of the AdaptMap dataset. Legend: ABR = *Abergelle*; ALP = *Alpen*; ANG = *Angora*; ANK = *Ankara*; ARG = *Argentata*; ASP = *Aspromontana*; BAB = *Barbari*; BAW = *Balaka*-*Ulongwe*; BEY = *Bermeya*; BIO = *Bionda*_*dell’Adamello;* BLB = *Bilberry*; BOE = *Boer*; BR I = *Bari*; BRK = *Barki*; BUR = *Burundi*_*goat*; BUT = *Bugituri*; CAM = *Cameroon_goat*; CAN = *Caninde’*; CAS = *Cashmere*; CCG = *Ciociara_Grigia*; CRE = *Creole*; CRP = *Carpatian*; CRS = *Corse*; DDP = *Dera Din Panah*; DIA = *Diana*; DIT = *Di_Teramo*; DJA = *Djallonke*; DZD = *Dedza*; FSS = *Fosses*; GAL = *Galla*; GAR = *Garganica*; GGT = *Girgentana*; GOG = *Gogo*; GUE = *Guera*; GUM = *Gumez*; ICL = *Icelandic*; JAT = *Jattan*; JON = *Jonica*; KAC = *Kachan*; KAM = *Kamori*; KAR = *Karamonja*; KEF = *Keffa*; KES =* Koh*-*e*-*sulmani*; KIK = *Kiko*; KIL = *Kil*; KLS = *Kilis*; LND = *Landin*; LNR = *Landrace*_*goat*; LOH = *Lohri*; LOP = *Local_Pothohari*; MAA = *Maasai*; MAL = *Mallorquina*; MAU = *Maure*; MEN = *Menabe*; MLG = *Malaguena*; MLS = *Maltese*_*Sarda*; MLT = *Maltese*; MLY = *Malya*; MOR = *Moroccan_goat*; MOX = *Moxoto’*; MSH = *Mashona*; MTB = *Matebele*; MUB = *Mubende*; MUG = *Murciano*-*Granadina*; NAI = *Naine*; NBN = *Nubian*; NGD = *Nganda*; NIC = *Nicastrese*; NRW = *Norwegian*; OIG = *Old_Irish_goat*; ORO = *Orobica*; OSS = *Oasis*; PAH = *Pahari*; PAL = *Palmera*; PAT = *Pateri*; PEU = *Peulh*; PRW = *Pare_White*; PTV = *Poitevine*; PVC = *Provencale*; PYR = *Pyrenean*; RAN = *Rangeland*; RAS = *Blanca_de_Rasquera*; RME = *Rossa_Mediterranea*; RSK = *Red_Sokoto*; SAA = *Saanen*; SAH = *Sahel*; SAR = *Sarda*; SDN = *Soudanaise*; SEA = *Small_East_Africa*; SEB = *Sebei*; SHL = *Sahel*; SID = *Saidi*; SNJ = *Sonjo*; SOF = *Sofia*; SPA = *Spanish*; TAP = *Tapri*; TAR = *Targui*; TED = *Teddi*; THA = *Thari*; TOG = *Toggenburg*; TUN = *Tunisian*; VAL = *Valdostana*; VSS = *Valpassiria*; WAD = *West_African_goat*; WYG = *Woyito_Guji*.

**Additional file 3.** Comparison of ROH across the breeds raised in different countries. The higher the value on the y axis, the bigger is the difference. The threshold of H = 5 is indicated with a red line.
