# Supplementary material for: Genome-wide patterns of homozygosity provide clues about the population history and adaptation of goats
Source: Genet Sel Evol. 2018 Nov 19;50:59. doi: 10.1186/s12711-018-0424-8 (PMC6241033; doi:10.1186/s12711-018-0424-8)
Supplement: Supplementary file 1 — Additional file 1: Table S1. Animals used for the analyses. Breed symbol, name and number (N). The country in which samples were collected (Country), the continental and subcontinental groups used for the analyses are reported. Table S2. Average fraction of the genome that contains ROH in each one of the breeds under analysis. Breed code (Breed) and average fraction of the genome that contains ROH (FROH). Breeds are reported based on FROH increasing values. Table S3. Summary statistics of number of ROH regions and genome coverage considering the sub-geographical and continental classification. min: minimum number of ROH regions or coverage detected; max: minimum number of ROH regions or coverage detected; mean: average number of ROH regions or coverage detected; sd: standard deviation from the mean value. Table S4. Comparison of pairwise least square means of the sub-geographical comparisons. Comparison: pairwise comparison considered; estimate: estimated difference in LSM; p.value: adjusted Bonferroni P value. Table S5. Summary statistics of number of ROH regions and genome coverage for comparison 1 and pairwise least square means comparison. min: minimum number of ROH regions or coverage detected; max: maximum number of ROH regions or coverage detected; mean: average number of ROH regions or coverage detected; sd: standard deviation from the mean value; estimate: estimated difference in LSM; p.value: adjusted Bonferroni P-value. Table S6. Chromosomal regions with a high level of homozygosity (the top 0.998 percentile of at least three consecutive SNPs) and overlaps (partial or complete) across continents and continental sub-divisions. NP = not present; “-“: no overlap detected; for the regions shared by most of the subgroups (All), the symbol of the genes detected within those regions are reported. See the bold number in the “overlap with other continental/sub-continental” column. (1) MIR217; MIR216B; CFAP36; PNPT1; PPP4R3B; EFEMP1. (2) GJB6; SAP18; MRPL57; ATP12A [file 12711_2018_424_MOESM1_ESM.doc]

**Supplementary Tables**

**Genome-wide patterns of homozygosity provide clues about the population history and adaptation of goats**

Francesca Bertolini, Tainã Figueiredo Cardoso, Gabriele Marras, Ezequiel L Nicolazzi, Max F Rothschild, Marcel Amills and the ADAPTMAP consortium.

**Additional file 1 Table S1**

Title: Animals used for the analyses

Description: Breed symbol, name and number (N.). Country of collection of the samples (Country), continental and subcontinental groups used for the analyses are reported.

| **BREED SYMBOL** | **Breed name** | **Country** | **N.** | Continental_group | Subcontinental group |
| --- | --- | --- | --- | --- | --- |
| ABR | Abergelle | Ethiopia | 49 | Africa | East Africa |
| ALP | Alpen | Switzerland | 50 | Europe | Central Europe |
|  |  | France | 50 |  |  |
|  |  | Italy | 50 |  |  |
| ANG | Angora | Agrentina | 50 | Asia | Near East |
|  |  | France | 26 |  |  |
|  |  | South Africa | 48 |  |  |
| ANK | Ankara | Turkey | 18 | Asia | Near East |
| ARG | Argentata | Italy | 24 | Europe | South Europe |
| ASP | Aspromontana | Italy | 23 | Europe | South Europe |
| BAB | Barbari | Pakistan | 16 | Asia | Central Asia |
| BAW | Balaka-Ulongwe | Malawi | 12 | Africa | East Africa |
| BEY | Bermeya | Spain | 23 | Europe | South Europe |
| BIO | Bionda_dell'Adamello | Italy | 24 | Europe | South Europe |
| BLB | Bilberry | Ireland | 10 | Europe | North Europe |
| BOE | Boer | Australia | 32 | Africa | South Africa |
|  |  | Switzerland | 50 |  |  |
|  |  | New Zealand | 13 |  |  |
|  |  | Usa | 29 |  |  |
|  |  | Zimbabwe | 17 |  |  |
| BOEx | Admixed_Boer | mixed countries | 7 | - | - |
| BRI | Bari | Pakistan | 25 | Asia | Central Asia |
| BRK | Barki | Egypt | 50 | Africa | North Africa |
| BUR | Burundi_goat | Burundi | 50 | Africa | Central West Africa |
| BUT | Bugituri | Pakistan | 31 | Asia | Central Asia |
| CAM | Cameroon_goat | Cameroon | 37 | Africa | Central West Africa |
| CAN | Caninde' | Brazil | 23 | South_America | - |
| CAS | Cashmere | Australia | 44 | Oceania | - |
| CCG | Ciociara_Grigia | Italy | 16 | Europe | South Europe |
| CRE | Creole | Argentina | 50 | South_America | - |
| CRP | Carpatian | Romania | 14 | Europe | Central Europe |
| CRS | Corse | France | 29 | Europe | Central Europe |
| DDP | Dera Din Panah | Pakistan | 20 | Asia | Central Asia |
| DIA | Diana | Madagascar | 14 | Africa | South Africa |
| DIT | Di_Teramo | Italy | 19 | Europe | South Europe |
| DJA | Djallonke | Burkina Faso | 10 | Africa | East Africa |
| DZD | Dedza | Malawi | 15 | Africa | South Africa |
| FSS | Fosses | France | 24 | Europe | Central Europe |
| GAL | Galla | Kenya | 23 | Africa | East Africa |
| GALxSAA | GallaxSaanen | Kenya | 1 | - | - |
| GAR | Garganica | Italy | 15 | Europe | South Europe |
| GGT | Girgentana | Italy | 24 | Europe | South Europe |
| GOG | Gogo | Tanzania | 12 | Africa | East Africa |
| GUE | Guera | Mali | 16 | Africa | Central West Africa |
| GUM | Gumez | Ethiopia | 39 | Africa | East Africa |
| ICL | Icelandic | Iceland | 11 | Europe | North Europe |
| JAT | Jattan | Pakistan | 15 | Asia | Central Asia |
| JON | Jonica | Italy | 11 | Europe | South Europe |
| KAC | Kachan | Pakistan | 19 | Asia | Central Asia |
| KAM | Kamori | Pakistan | 38 | Asia | Central Asia |
| KAR | Karamonja | Uganda | 19 | Africa | East Africa |
| KEF | Keffa | Ethiopia | 44 | Africa | East Africa |
| KES | Koh-e-sulmani | Pakistan | 13 | Asia | Central Asia |
| KIK | Kiko | Usa | 11 | Oceania | - |
| KIL | Kil | Turkey | 23 | Asia | Near East |
| KLS | Kilis | Turkey | 36 | Asia | Near East |
| LND | Landin | Mozambique | 29 | Africa | South Africa |
| LNR | Landrace_goat | mixed countries | 85 | Europe | North Europe |
| LOH | Lohri | Pakistan | 17 | Asia | Central Asia |
| LOP | Local_Pothohari | Pakistan | 13 | Asia | Central Asia |
| MAA | Maasai | Tanzania | 18 | Africa | East Africa |
| MAL | Mallorquina | Spain | 18 | Europe | South Europe |
| MAU | Maure | Mali | 13 | Africa | East Africa |
| MEN | Menabe | Madagascar | 19 | Africa | South Africa |
| MLG | Malaguena | Spain | 40 | Europe | South Europe |
| MLS | Maltese_Sarda | Italy | 12 | Europe | South Europe |
| MLT | Maltese | Italy | 16 | Europe | South Europe |
| MLY | Malya | mixed countries | 23 | Africa | East Africa |
| MOR | Moroccan_goat | Morocco | 10 | Africa | North Africa |
| MOX | Moxoto' | Brazil | 23 | South_America | - |
| MSH | Mashona | Zimbabwe | 22 | Africa | South Africa |
| MTB | Matebele | Zimbabwe | 22 | Africa | South Africa |
| MTBx | Matabele_cross | Zimbabwe | 24 | - | - |
| MUB | Mubende | Uganda | 18 | Africa | East Africa |
| MUBx | Admixed | Uganda | 3 | - | - |
| MUG | Murciano-Granadina | Spain | 20 | Europe | South Europe |
| NAI | Naine | Mali | 14 | Africa | East Africa |
| NBN | Nubian | Argentina | 13 | Africa | North Africa |
|  |  | Egypt | 50 |  |  |
| NGD | Nganda | Argentina | 11 | Africa | East Africa |
| NIC | Nicastrese | Egypt | 20 | Europe | South Europe |
| NRW | Norwegian | Tanzania | 17 | Europe | North Europe |
| OIG | Old_Irish_goat | Ireland | 13 | Europe | North Europe |
| OIGx | Old_Irish_goat_cross | Ireland | 10 | - | - |
| ORO | Orobica | Italy | 22 | Europe | South Europe |
| OSS | Oasis | Egypt | 50 | Africa | North Africa |
| PAH | Pahari | Pakistan | 19 | Asia | Central Asia |
| PAL | Palmera | Spain | 15 | Africa | North Africa |
| PAT | Pateri | Pakistan | 27 | Asia | Central Asia |
| PEU | Peulh | Mali | 22 | Africa | Central West Africa |
| PRW | Pare_White | Tanzania | 19 | Africa | East Africa |
| PTV | Poitevine | France | 27 | Europe | Central Europe |
| PVC | Provencale | France | 17 | Europe | Central Europe |
| PYR | Pyrenean | France | 26 | Europe | Central Europe |
| RAN | Rangeland | Australia | 50 | Oceania | - |
| RAS | Blanca_de_Rasquera | Spain | 20 | Europe | South Europe |
| RME | Rossa_Mediterranea | Italy | 30 | Europe | South Europe |
| RSK | Red_Sokoto | Nigeria | 19 | Africa | Central West Africa |
| SAA | Saanen | Argentina | 16 | Europe | Central Europe |
|  |  | Switzerland | 41 |  |  |
|  |  | France | 50 |  |  |
|  |  | Italy | 23 |  |  |
|  |  | Tanzania | 15 |  |  |
| SAAxANB | SaanenxAnglo_NubianF2 | Brazil | 14 | - | - |
| SAAxCRE | SaanenxCreole | Argentina | 19 | - | - |
| SAH | Sahel | Burkina Faso | 15 | Africa | Central West Africa |
| SAR | Sarda | Italy | 27 | Europe | South Europe |
| SDN | Soudanaise | Mali | 22 | Africa | Central West Africa |
| SEA | Small_East_Africa | mixed countries | 50 | Africa | East Africa |
| SEAx | Admixed_Small_East_Africa | Kenya | 2 | - | - |
| SEAxALP | Small_East_AfricaxAlpine | Kenya | 3 | - | - |
| SEAxGAL | Small_East_AfricaxGalla | Kenya | 18 | - | - |
| SEAxSAA | Small_East_AfricaxSaanen | Kenya | 2 | - | - |
| SEAxTOG | Small_East_AfricaxToggenburg | Kenya | 5 | - | - |
| SEB | Sebei | Uganda | 21 | Africa | East Africa |
| SHL | Sahel | Nigeria | 19 | Africa | Central West Africa |
| SID | Saidi | Egypt | 50 | Africa | North Africa |
| SNJ | Sonjo | Tanzania | 20 | Africa | East Africa |
| SOF | Sofia | Madagascar | 22 | Africa | South Africa |
| SPA | Spanish | Usa | 17 | South_America | - |
| TAP | Tapri | Pakistan | 22 | Asia | Central Asia |
| TAR | Targui | Mali | 19 | Africa | Central West Africa |
| TED | Teddi | Pakistan | 47 | Asia | Central Asia |
| THA | Thari | Pakistan | 16 | Asia | Central Asia |
| TOG | Toggenburg | mixed countries | 20 | Europe | Central Europe |
| TUN | Tunisian | Tunisia | 21 | Africa | North Africa |
| VAL | Valdostana | Italy | 24 | Europe | South Europe |
| VSS | Valpassiria | Italy | 24 | Europe | Central Europe |
| WAD | West_African_goat | mixed countries | 50 | Africa | Central West Africa |
| WYG | Woyito_Guji | Ethiopia | 39 | Africa | East Africa |

**Additional file 1 Table S2**

Title**:** Average fraction of the genome that contains ROH in each one of the breeds under analysis

Description: Breed code (Breed) and average fraction of the genome that contains ROH (FROH). Breeds are reported based on FROH increasing values

| **Breed** | ARG | CRP | GAL | MAU | KAR | PEU | BEY | SNJ | SDN | SEB | MLG | BRK | KIL | WYG | GUM | KLS | MAA | TUN | SHL | NGD | ABR |
| --- | --- | --- | --- | --- | --- | --- | --- | --- | --- | --- | --- | --- | --- | --- | --- | --- | --- | --- | --- | --- | --- |
| **F_ROH_** | 0.02 | 0.02 | 0.02 | 0.02 | 0.03 | 0.03 | 0.03 | 0.03 | 0.03 | 0.03 | 0.03 | 0.03 | 0.03 | 0.03 | 0.03 | 0.03 | 0.03 | 0.03 | 0.04 | 0.04 | 0.04 |
| **Breed** | GOG | CRS | TAR | SAR | CAM | THA | RME | SAH | BIO | JON | DJA | RAN | NAI | MUG | ANK | KIK | ASP | VSS | SID | PAH | KES |
| **F_ROH_** | 0.04 | 0.04 | 0.04 | 0.04 | 0.04 | 0.04 | 0.04 | 0.04 | 0.05 | 0.05 | 0.05 | 0.05 | 0.05 | 0.05 | 0.05 | 0.05 | 0.05 | 0.06 | 0.06 | 0.06 | 0.06 |
| **Breed** | BUR | MTB | GAR | RSK | SPA | LOP | CCG | GUE | PVC | SEA | NIC | WAD | MUB | PRW | KEF | ALP | MOR | NRW | SAA | FSS | OSS |
| **F_ROH_** | 0.06 | 0.06 | 0.06 | 0.06 | 0.06 | 0.06 | 0.06 | 0.06 | 0.06 | 0.07 | 0.07 | 0.07 | 0.07 | 0.07 | 0.07 | 0.07 | 0.08 | 0.08 | 0.08 | 0.09 | 0.10 |
| **Breed** | LOH | RAS | ANG | CAS | DIT | PYR | MLS | TOG | NBN | CRE | PTV | BAW | VAL | TED | MSH | JAT | MLY | TAP | MAL | ORO | PAT |
| **F_ROH_** | 0.10 | 0.10 | 0.10 | 0.10 | 0.11 | 0.11 | 0.12 | 0.12 | 0.12 | 0.12 | 0.12 | 0.12 | 0.12 | 0.13 | 0.13 | 0.13 | 0.13 | 0.14 | 0.14 | 0.14 | 0.14 |
| **Breed** | OIG | GGT | MLT | DDP | LNR | DZD | BUT | MOX | LND | CAN | BRI | BOE | BAB | BLB | PAL | KAM | KAC | DIA | MEN | SOF | ICL |
| **F_ROH_** | 0.14 | 0.15 | 0.15 | 0.16 | 0.16 | 0.17 | 0.17 | 0.18 | 0.18 | 0.18 | 0.20 | 0.20 | 0.21 | 0.22 | 0.23 | 0.25 | 0.25 | 0.26 | 0.32 | 0.35 | 0.66 |

**Additional file 1 Table S3**

Title: Summary statistics of number of ROH regions and genome coverage considering the sub-geographical and continental classification.

Description:

min: minimum number of ROH regions or coverage detected.

max: minimum number of ROH regions or coverage detected.

mean: average number of ROH regions or coverage detected.

sd: standard deviation from the mean value.

|  | ROH regions (N.) | | | ROH coverage (Mb) | | |
| --- | --- | --- | --- | --- | --- | --- |
|  | min | max | mean (±stdev) | min | max | mean (±stdev) |
| America | 7 | 384 | 136 (74) | 7.57 | 833.73 | 333.83 (198.26) |
| Oceania | 11 | 84 | 49 (14) | 16.22 | 651.76 | 182.19 (118.25) |
| Central Asia | 18 | 179 | 90 (34) | 21.55 | 1,269.48 | 370.15 (260.64) |
| Near East | 9 | 262 | 68 (45) | 10.87 | 893.67 | 256.29 (210.11) |
| **Asia** | 9 | 262 | 82 (40) | 10.87 | 1,269.48 | 326.48 (248.67) |
| Central Europe | 3 | 147 | 51 (25) | 5.80 | 957.07 | 194.90 (146.76) |
| North Europe | 6 | 454 | 98 (84) | 6.81 | 1,978.28 | 479.17 (399.19) |
| South Europe | 5 | 159 | 49 (31) | 7.9 | 938.86 | 183.47 (179.09) |
| **Europe** | 3 | 454 | 56 (43) | 5.80 | 1978.28 | 277.37 (230.89) |
| Central Western Africa | 24 | 140 | 62 (27) | 28.76 | 855.59 | 122.16 (119.59) |
| East Africa | 16 | 263 | 59 (28) | 24.14 | 927.72 | 127.16 (134.54) |
| North Africa | 9 | 315 | 61 (60) | 12.47 | 1,096.99 | 206.96 (209.35) |
| South Africa | 47 | 425 | 184 (91) | 110.73 | 1,279.294 | 504.35 (223.71) |
| **Africa** | 9 | 427 | 89 (76) | 12.47 | 1,279.30 | 229.00 (231.16) |

**Additional file 1 Table S4**

Title: pairwise least square means comparison of the the sub-geographical comparisons

Description:

Comparison: pairwise comparison considered

estimate: estimate difference in LSM

p.value: Adjusted Bonferroni P-value.

|  | ROH Regions (N.) | | ROH coverage (Mb) | |
| --- | --- | --- | --- | --- |
| Comparison | estimate | p.value | estimate | p.value |
| America-Central_Asia | 46.16 | <.0001 | -36.32 | 1.0000 |
| America-Central_Europe | 85.42 | <.0001 | 138.93 | <.0001 |
| America-Central_Western_Africa | 74.02 | <.0001 | 211.67 | <.0001 |
| America-East_Africa | 77.29 | <.0001 | 206.67 | <.0001 |
| America-Near_East | 68.30 | <.0001 | 77.55 | 0.0584 |
| America-North_Africa | 75.06 | <.0001 | 126.87 | <.0001 |
| America-North_Europe | 38.62 | <.0001 | -145.34 | 0.0108 |
| America-Oceania | 87.07 | <.0001 | 151.64 | <.0001 |
| America-South_Africa | -47.88 | <.0001 | -170.52 | <.0001 |
| America-South_Europe | 87.38 | <.0001 | 150.36 | <.0001 |
| Central_Asia-Central_Europe | 39.26 | <.0001 | 175.25 | <.0001 |
| Central_Asia-Central_Western_Africa | 27.86 | <.0001 | 247.98 | <.0001 |
| Central_Asia-East_Africa | 31.13 | <.0001 | 242.99 | <.0001 |
| Central_Asia-Near_East | 22.14 | <.0001 | 113.86 | <.0001 |
| Central_Asia-North_Africa | 28.90 | <.0001 | 163.19 | <.0001 |
| Central_Asia-North_Europe | -7.54 | 1.0000 | -109.03 | 0.1803 |
| Central_Asia-Oceania | 40.91 | <.0001 | 187.96 | <.0001 |
| Central_Asia-South_Africa | -94.04 | <.0001 | -134.20 | <.0001 |
| Central_Asia-South_Europe | 41.22 | <.0001 | 186.68 | <.0001 |
| Central_Europe-Central_Western_Africa | -11.40 | 0.0955 | 72.74 | <.0001 |
| Central_Europe-East_Africa | -8.13 | 0.5156 | 67.74 | <.0001 |
| Central_Europe-Near_East | -17.12 | 0.0009 | -61.39 | 0.0073 |
| Central_Europe-North_Africa | -10.37 | 0.2694 | -12.06 | 1.0000 |
| Central_Europe-North_Europe | -46.80 | <.0001 | -284.27 | <.0001 |
| Central_Europe-Oceania | 1.65 | 1.0000 | 12.71 | 1.0000 |
| Central_Europe-South_Africa | -133.31 | <.0001 | -309.45 | <.0001 |
| Central_Europe-South_Europe | 1.96 | 1.0000 | 11.43 | 1.0000 |
| Central_Western_Africa-East_Africa | 3.27 | 1.0000 | -4.99 | 1.0000 |
| Central_Western_Africa-Near_East | -5.72 | 1.0000 | -134.12 | <.0001 |
| Central_Western_Africa-North_Africa | 1.03 | 1.0000 | -84.80 | <.0001 |
| Central_Western_Africa-North_Europe | -35.40 | <.0001 | -357.01 | <.0001 |
| Central_Western_Africa-Oceania | 13.05 | 0.9599 | -60.02 | 0.0006 |
| Central_Western_Africa-South_Africa | -121.91 | <.0001 | -382.19 | <.0001 |
| Central_Western_Africa-South_Europe | 13.36 | 0.0177 | -61.31 | <.0001 |
| East_Africa-Near_East | -8.99 | 1.0000 | -129.13 | <.0001 |
| East_Africa-North_Africa | -2.23 | 1.0000 | -79.80 | <.0001 |
| East_Africa-North_Europe | -38.67 | <.0001 | -352.02 | <.0001 |
| East_Africa-Oceania | 9.78 | 1.0000 | -55.03 | 0.0016 |
| East_Africa-South_Africa | -125.17 | <.0001 | -377.19 | <.0001 |
| East_Africa-South_Europe | 10.09 | 0.0932 | -56.31 | <.0001 |
| Near_East-North_Africa | 6.75 | 1.0000 | 49.33 | 0.6380 |
| Near_East-North_Europe | -29.68 | <.0001 | -222.89 | <.0001 |
| Near_East-Oceania | 18.77 | 0.0562 | 74.10 | 0.0038 |
| Near_East-South_Africa | -116.19 | <.0001 | -248.06 | <.0001 |
| Near_East-South_Europe | 19.08 | 0.0001 | 72.81 | 0.0010 |
| North_Africa-North_Europe | -36.44 | <.0001 | -272.21 | <.0001 |
| North_Africa-Oceania | 12.01 | 1.0000 | 24.77 | 1.0000 |
| North_Africa-South_Africa | -122.94 | <.0001 | -297.39 | <.0001 |
| North_Africa-South_Europe | 12.33 | 0.0569 | 23.49 | 1.0000 |
| North_Europe-Oceania | 48.45 | <.0001 | 296.99 | <.0001 |
| North_Europe-South_Africa | -86.50 | <.0001 | -25.18 | 1.0000 |
| North_Europe-South_Europe | 48.76 | <.0001 | 295.70 | <.0001 |
| Oceania-South_Africa | -134.95 | <.0001 | -322.16 | <.0001 |
| Oceania-South_Europe | 0.31 | 1.0000 | -1.28 | 1.0000 |
| South_Africa-South_Europe | 135.27 | <.0001 | 320.88 | <.0001 |

**Additional file 1 Table S5**

Title: Summary statistics of number of ROH regions and genome coverage of the comparison 1 and pairwise least square means comparison.

Description:

min: minimum number of ROH regions or coverage detected.

max: minimum number of ROH regions or coverage detected.

mean: average number of ROH regions or coverage detected.

sd: standard deviation from the mean value.

estimate: estimate difference in LSM

p.value: Adjusted Bonferroni P-value.

|  |  | ROH Regions (N.) | | | | | ROH coverage (Mb) | | | | |
| --- | --- | --- | --- | --- | --- | --- | --- | --- | --- | --- | --- |
| group | N | min | max | mean(±stdev) | estimate | p.value | min | max | mean(±stdev) | estimate | p.value |
| Mixed | 102 | 6 | 194 | 62(43) | -19.65 | <.0001 | 7.21 | 1269.93 | 162.52(195.23) | -126.76 | <.0001 |
| Pure | 926 | 3 | 250 | 82(47) |  |  | 5.69 | 888.73 | 289.27(194.39) |  |  |
| Large | 261 | 9 | 181 | 46(31) | -9.05 | 0.001 | 11.19 | 701.23 | 154.2(132.52) | -58.48 | <.0001 |
| Small | 243 | 3 | 150 | 55(30) |  |  | 3.61 | 893.3 | 212.68(178.38) |  |  |
| Improved | 547 | 3 | 188 | 104(46) | 48.49 | <.0001 | 5.69 | 888.71 | 377.40(190.05) | 114.86 | <.0001 |
| Traditional | 427 | 10 | 129 | 55(23) |  |  | 15,87 | 1153,62 | 262.54(208.88) |  |  |

**Additional file 1 Table S6**

Title: Chromosomal region of high homozygosity (the top 0.998 percentile of at least 3 consecutive SNPs) and overlap (partial of complete) across continents and continental sub-divisions.

Description: NP=not present.

“-“: no overlap detected.

For the regions shared by the majorities of the subgroups (All), genes symbol within those regions are reported. See the bold number in the “overlap with other continental/sub-continental” column

1. MIR217; MIR216B; CFAP36; PNPT1; PPP4R3B; EFEMP1
2. GJB6; SAP18; MRPL57; ATP12A; CENPJ; MPHOSPH8; ZMYM5; GJA3; GJB2; CRYL1; IL17D; EEF1AKMT1; LATS2; SKA3; ZDHHC20; FGF9; RNF17; PSPC1; ZMYM2; IFT88; XPO4; MICU2; PARP4
3. MAB21L1; DCLK1; NBEA
4. TPPP3; AGRP; CARMIL2; PARD6A; ENKD1; C18H16orf86; TSNAXIP1; THAP11; NUTF2; EDC4; NRN1L; LCAT; DPEP3; DPEP2; DDX28; SLC7A6OS; LRRC36; ZDHHC1; ATP6V0D1; FAM65A; ACD; GFOD2; CENPT; PSKH1; PSMB10; DUS2; ESRP2; PLA2G15; SLC7A6; SMPD3; HSD11B2; CTCF; RANBP10; NFATC3; PRMT7; SLC12A4

| **Continent** | **chr** | **start** | **end** | **length (Kb)** | **overlap with sub-continental division** | **overlap with other continental/sub-continental** |
| --- | --- | --- | --- | --- | --- | --- |
| Europe | 11 | 37793580 | 38325859 | 532,28 | CentralEurope | All, Africa, America, EastAfrica |
|  | 12 | 43632105 | 44611526 | 979,42 | SouthEurope, CentralEurope | America_2, All, NorthAfrica, SouthAfrica |
|  | 12 | 50018490 | 51283345 | 1.264,86 | SouthEurope, CentralEurope | SouthAfrica, Oceania, America_2, All, Africa, Asia, EastAfrica_2, CentralWestAfrica, NorthAfrica |
|  | 12 | 60220203 | 60950668 | 730,47 | NorthEurope, SouthEurope | Oceania, All, Africa, CentralAsia, Asia, EastAfrica_2, CentralWestAfrica, NorthAfrica |
|  | 18 | 36283544 | 36966108 | 682,56 | NorthEurope, CentralEurope, SouthEurope | All, Africa, CentralAsia, Asia, EastAfrica_2, CentralWestAfrica |
| Asia | 6 | 70496152 | 71044838 | 548,69 | NearEast | - |
|  | 13 | 62900048 | 63786338 | 886,29 | CentralAsia | CentralEurope |
|  | 12 | 50327948 | 50553986 | 226,04 | - | Oceania, America, All, Africa, EastAfrica, CentralWestAfrica, CentralEurope, NorthAfrica, Europe, SouthEurope |
|  | 12 | 60112675 | 60950668 | 837,99 | CentralAsia | Oceania, All, Africa, EastAfrica, CentralWestAfrica, NorthAfrica, Europe, NorthEurope, SouthEurope |
|  | 18 | 14647245 | 16378467 | 1.731,22 | CentralAsia, NearEast | - |
|  | 18 | 36448079 | 37201445 | 753,37 | CentralAsia | SouthEurope, All, Africa, EastAfrica, CentralWestAfrica, CentralEurope, Europe, NorthEurope |
| Africa | 11 | 37793580 | 38565140 | 771,56 | EastAfrica | All, Europe, America, CentralEurope |
|  | 12 | 50018490 | 51486716 | 1.468,23 | SouthAfrica, CentralWestAfrica, NorthAfrica, EastAfrica | Oceania, America, All, Asia, CentralEurope, Europe, SouthEurope, |
|  | 12 | 60080989 | 61130443 | 1.049,45 | CentralWestAfrica, NorthAfrica, EastAfrica | Oceania, All, CentralAsia, Asia, Europe, NorthEurope, SouthEurope |
|  | 18 | 36054355 | 37201445 | 1.147,09 | CentralWestAfrica, EastAfrica | SouthEurope, All, CentralAsia, Asia, CentralEurope, Europe, NorthEurope |
| America | 11 | 37665957 | 38186897 | 520,94 | NP | All, Africa, Europe, EastAfrica, CentralEurope |
|  | 12 | 43067469 | 44644892 | 1.577,42 | NP | All, CentralEurope, NorthAfrica, Europe, SouthAfrica, SouthEurope |
|  | 12 | 49396564 | 51379110 | 1.982,55 | NP | SouthAfrica, CentralWestAfrica, Oceania, All, Africa, Asia, EastAfrica, CentralWestAfrica, CentralEurope, NorthAfrica, Europe, SouthEurope |
|  | 18 | 26825850 | 27075372 | 249,52 | NP | - |
|  | 18 | 27494550 | 27668309 | 173,76 | NP | - |
| Oceania | 3 | 1,1E+08 | 111159499 | 1.007,13 | NP | - |
|  | 5 | 95666102 | 96541979 | 875,88 | NP | - |
|  | 7 | 59816579 | 59924105 | 107,53 | NP | - |
|  | 8 | 43939687 | 44623411 | 683,72 | NP | - |
|  | 11 | 94227241 | 94498221 | 270,98 | NP | - |
|  | 12 | 48302279 | 48444019 | 141,74 | NP | - |
|  | 12 | 50047466 | 50755554 | 708,09 | NP | SouthAfrica, America, All, Africa, Asia, EastAfrica, CentralWestAfrica, CentralEurope, NorthAfrica, Europe, SouthEurope |
|  | 12 | 60170203 | 60910543 | 740,34 | NP | All, Africa, CentralAsia, Asia, EastAfrica, CentralWestAfrica, NorthAfrica, Europe, NorthEurope, SouthEurope |
| All | 11 | 37793580 | 38325859 | 532,28 | Europe, Africa | EastAfrica, CentralEurope **(1)** |
|  | 12 | 43632105 | 44532383 | 900,28 | Europe | CentralEurope, NorthAfrica, SouthAfrica, SouthEurope |
|  | 12 | 50018490 | 51379110 | 1.360,62 | Africa, Oceania, Asia, Europe, America, Oceania | SouthAfrica, EastAfrica, CentralWestAfrica, CentralEurope, NorthAfrica, SouthEurope **(2)** |
|  | 12 | 60112675 | 61020537 | 907,86 | Europe, Africa, Oceania, Asia | CentralAsia, EastAfrica, CentralWestAfrica, NorthAfrica, NorthEurope, SouthEurope **(3)** |
|  | 18 | 36221683 | 37006519 | 784,84 | Europe, Africa, Asia | SouthEurope, CentralAsia, EastAfrica, CentralWestAfrica, CentralEurope, NorthEurope **(4)** |

**Additional file 1 Table S7**

Title: Chromosomal region of high divergence in homozygosity across the different countries in the same breeds.

Description: For each breed-based analyses, chromosome (chr), start and end of the regions with H≥5 are reported.

| Breed | Country | Chr | Start | End |
| --- | --- | --- | --- | --- |
| Alpen | Switzerland | 3 | 91540801 | 92291726 |
|  |  | 13 | 62900048 | 63686657 |
|  | Italy | 11 | 94319962 | 95442334 |
| Angora | South Africa | 14 | 53140977 | 53382745 |
|  |  | 23 | 20441 | 2297323 |
|  |  | 23 | 15058707 | 16807082 |
| Saanen | Switzerland | 6 | 29892079 | 30139296 |
|  | France | 13 | 50402269 | 51090982 |
| Nubian | - | 6 | 40875711 | 41385016 |
