# Supplementary figures and images for: Genome-wide patterns of homozygosity provide clues about the population history and adaptation of goats
Source: Genet Sel Evol. 2018 Nov 19;50:59. doi: 10.1186/s12711-018-0424-8 (PMC6241033; doi:10.1186/s12711-018-0424-8)

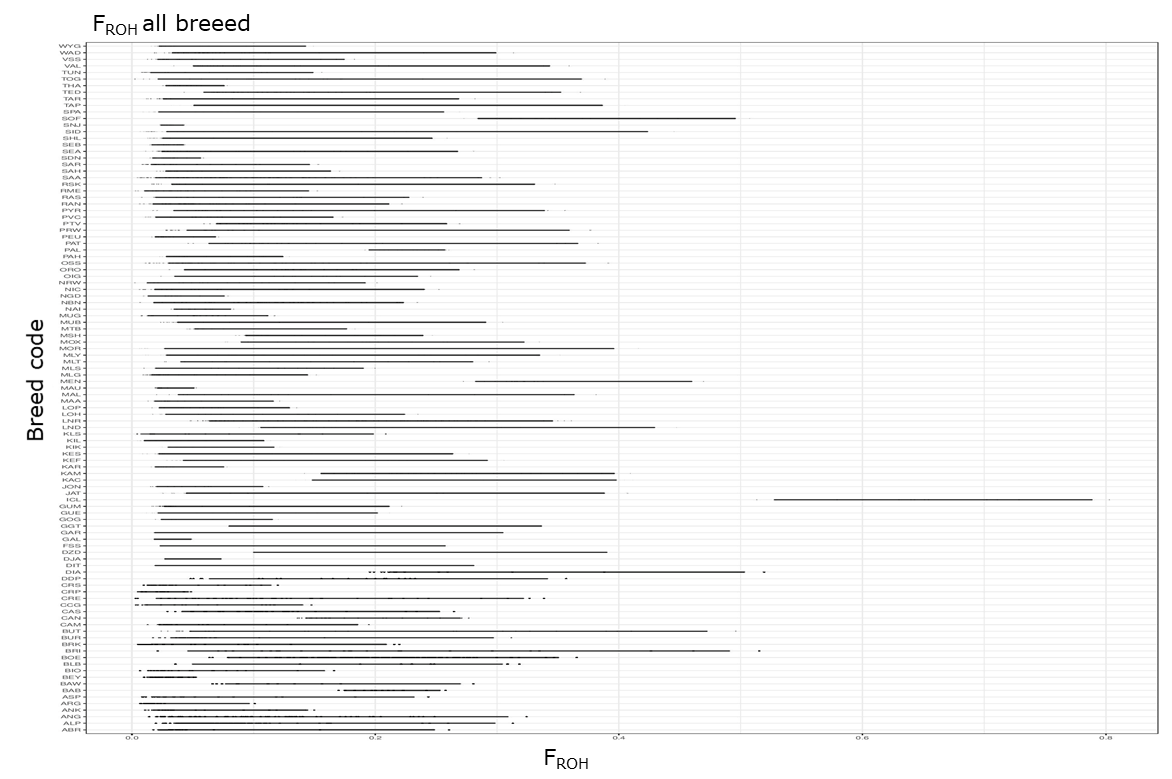

Supplement: Supplementary file 2 — Additional file 2: Figure S1. FROH calculated in the single breeds of the AdaptMap dataset. Legend: ABR = Abergelle; ALP = Alpen; ANG = Angora; ANK = Ankara; ARG = Argentata; ASP = Aspromontana; BAB = Barbari; BAW = Balaka-Ulongwe; BEY = Bermeya; BIO = Bionda_dell’Adamello; BLB = Bilberry; BOE = Boer; BR I = Bari; BRK = Barki; BUR = Burundi_goat; BUT = Bugituri; CAM = Cameroon_goat; CAN = Caninde’; CAS = Cashmere; CCG = Ciociara_Grigia; CRE = Creole; CRP = Carpatian; CRS = Corse; DDP = Dera Din Panah; DIA = Diana; DIT = Di_Teramo; DJA = Djallonke; DZD = Dedza; FSS = Fosses; GAL = Galla; GAR = Garganica; GGT = Girgentana; GOG = Gogo; GUE = Guera; GUM = Gumez; ICL = Icelandic; JAT = Jattan; JON = Jonica; KAC = Kachan; KAM = Kamori; KAR = Karamonja; KEF = Keffa; KES = Koh-e-sulmani; KIK = Kiko; KIL = Kil; KLS = Kilis; LND = Landin; LNR = Landrace_goat; LOH = Lohri; LOP = Local_Pothohari; MAA = Maasai; MAL = Mallorquina; MAU = Maure; MEN = Menabe; MLG = Malaguena; MLS = Maltese_Sarda; MLT = Maltese; MLY = Malya; MOR = Moroccan_goat; MOX = Moxoto’; MSH = Mashona; MTB = Matebele; MUB = Mubende; MUG = Murciano-Granadina; NAI = Naine; NBN = Nubian; NGD = Nganda; NIC = Nicastrese; NRW = Norwegian; OIG = Old_Irish_goat; ORO = Orobica; OSS = Oasis; PAH = Pahari; PAL = Palmera; PAT = Pateri; PEU = Peulh; PRW = Pare_White; PTV = Poitevine; PVC = Provencale; PYR = Pyrenean; RAN = Rangeland; RAS = Blanca_de_Rasquera; RME = Rossa_Mediterranea; RSK = Red_Sokoto; SAA = Saanen; SAH = Sahel; SAR = Sarda; SDN = Soudanaise; SEA = Small_East_Africa; SEB = Sebei; SHL = Sahel; SID = Saidi; SNJ = Sonjo; SOF = Sofia; SPA = Spanish; TAP = Tapri; TAR = Targui; TED = Teddi; THA = Thari; TOG = Toggenburg; TUN = Tunisian; VAL = Valdostana; VSS = Valpassiria; WAD = West_African_goat; WYG = Woyito_Guji. [file 12711_2018_424_MOESM2_ESM.tif]
