# Supplementary material for: Genome-wide patterns of homozygosity provide clues about the population history and adaptation of goats
Source: Genet Sel Evol. 2018 Nov 19;50:59. doi: 10.1186/s12711-018-0424-8 (PMC6241033; doi:10.1186/s12711-018-0424-8)

Alpine (ALP)

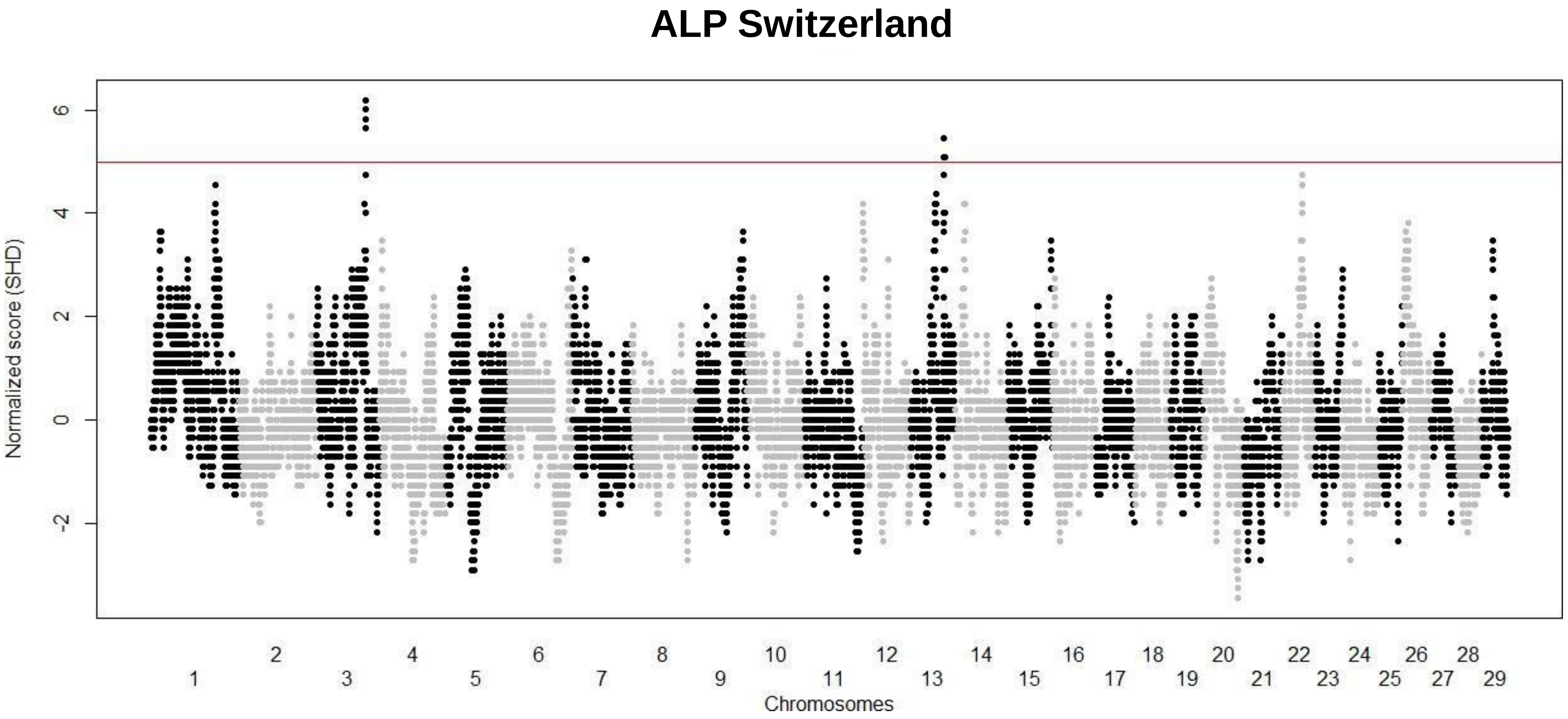

ALP Switzerland
Normalized score (SHD)
6
4
2
0
-2
1
2
3
4
5
6
7
8
9
10
11
12
13
14
15
16
17
18
19
20
21
22
23
24
25
26
27
28
29
Chromosomes

ALP France

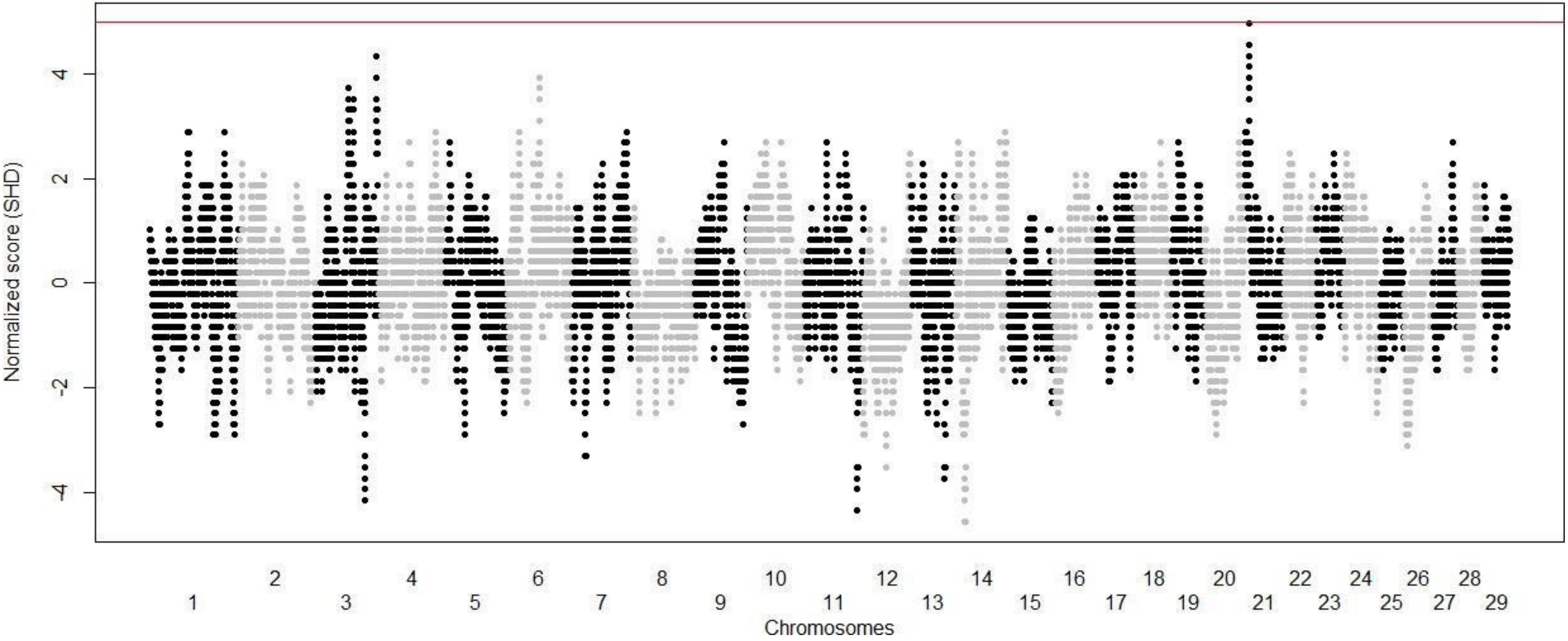

Normalized score (SHD)
4
2
0
-2
-4
2
4
6
8
10
12
14
16
18
20
22
24
26
28
1
3
5
7
9
11
13
15
17
19
21
23
25
27
29
Chromosomes

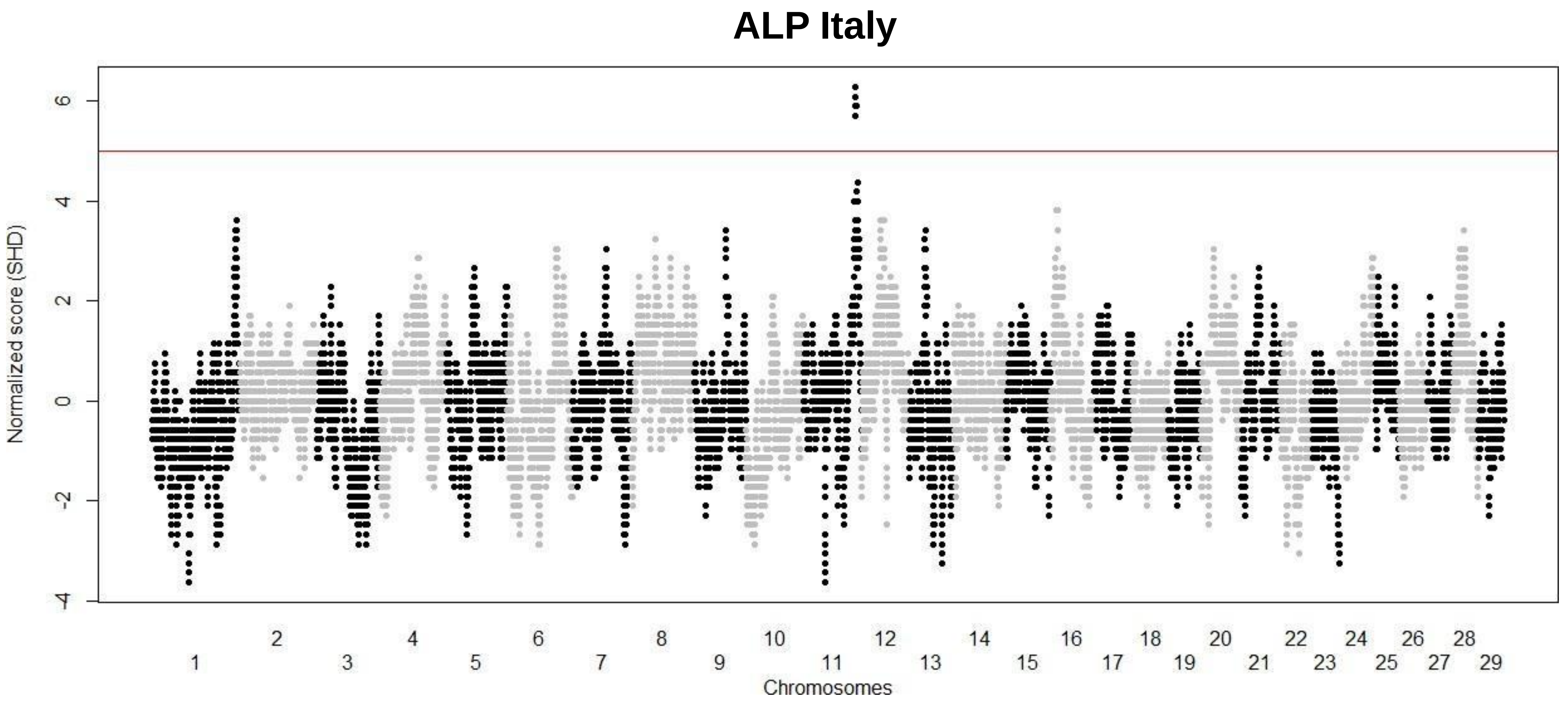
ALP Italy
Normalized score (SHD)
6
4
2
0
-2
-4
1 2 3 4 5 6 7 8 9 10 11 12 13 14 15 16 17 18 19 20 21 22 23 24 25 26 27 28 29
Chromosomes

Angora (ANG)

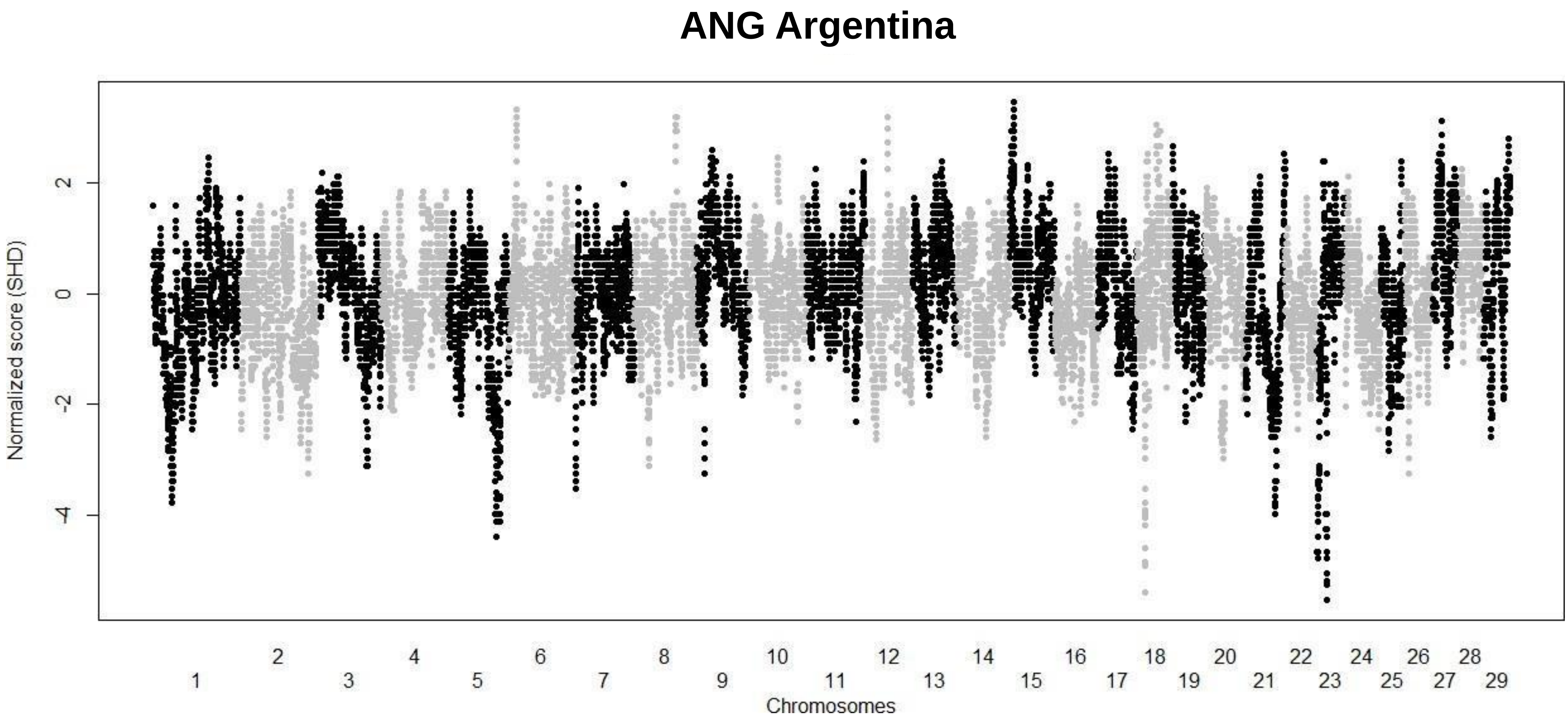
ANG Argentina
Normalized score (SHD)
2
0
-2
-4
1
2
3
4
5
6
7
8
9
10
11
12
13
14
15
16
17
18
19
20
21
22
23
24
25
26
27
28
29
Chromosomes

ANG France

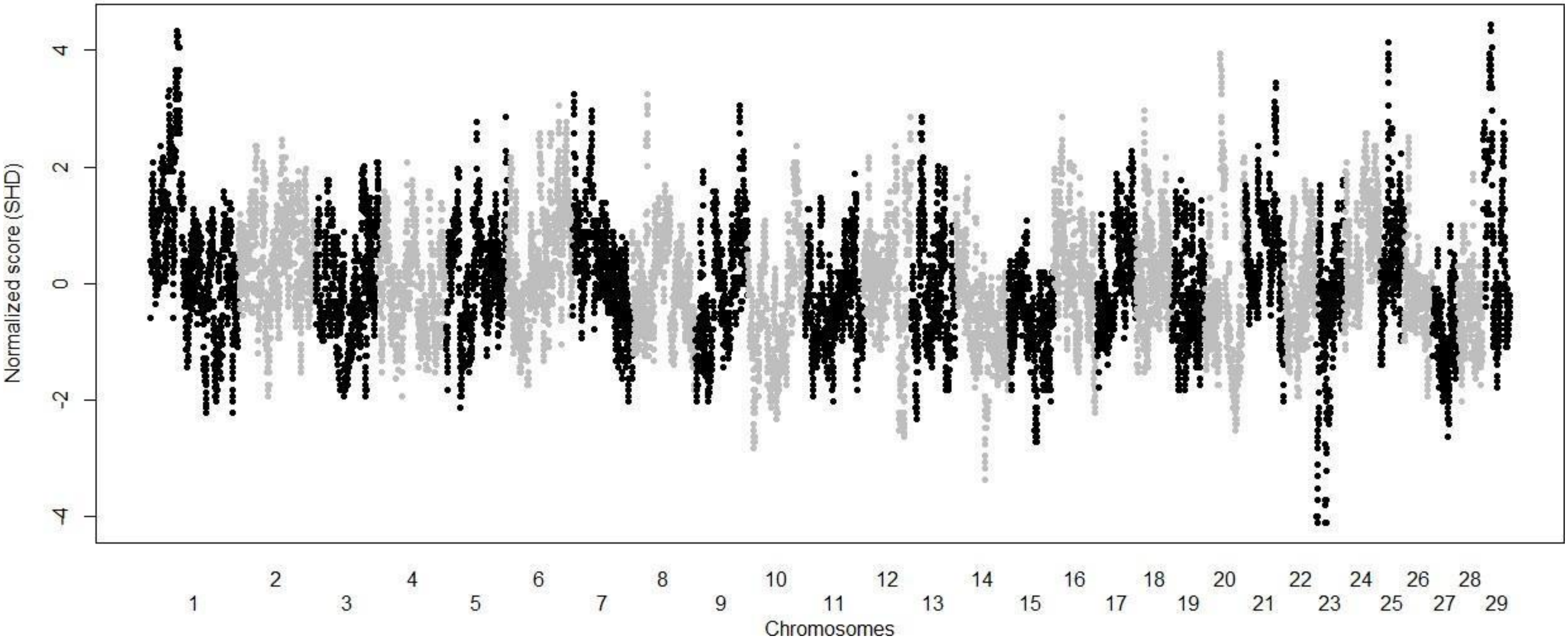

Normalized score (SHD)
4
2
0
-2
-4
1 2 3 4 5 6 7 8 9 10 11 12 13 14 15 16 17 18 19 20 21 22 23 24 25 26 27 28 29
Chromosomes

ANG South Africa

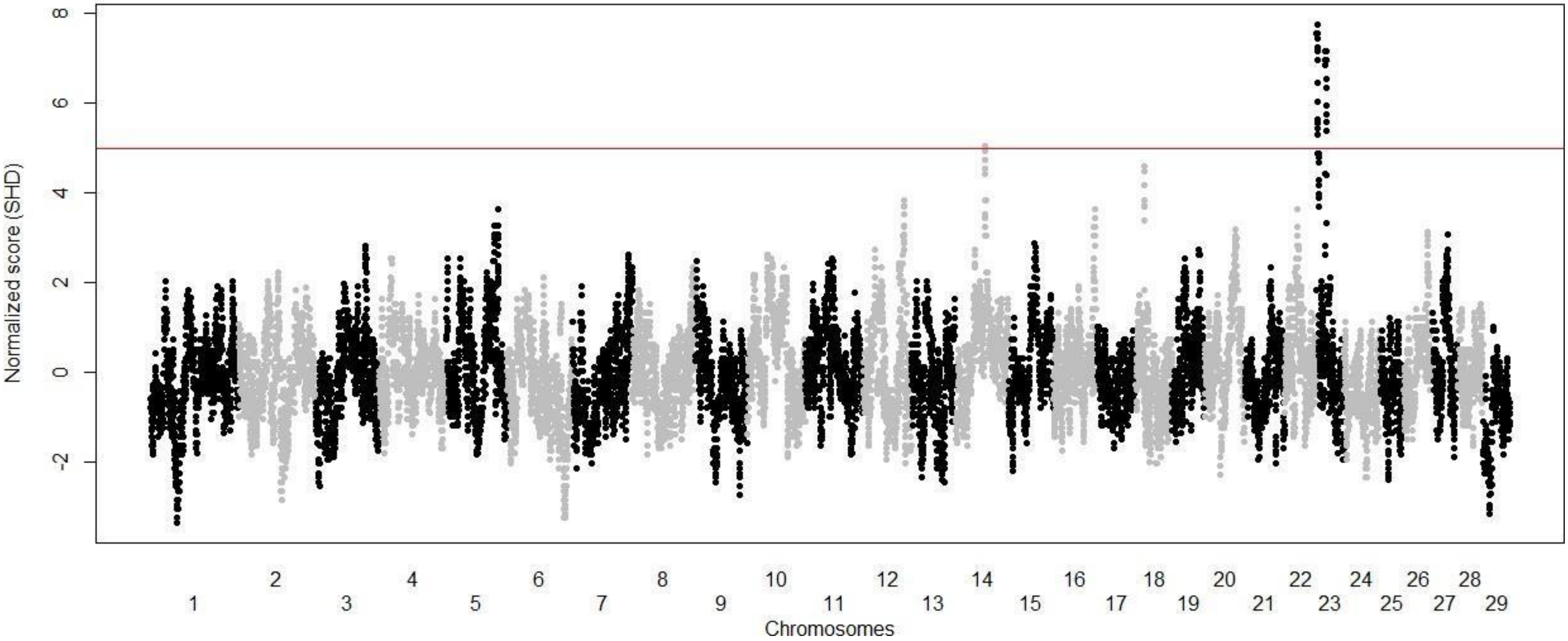

Boer (BOE)

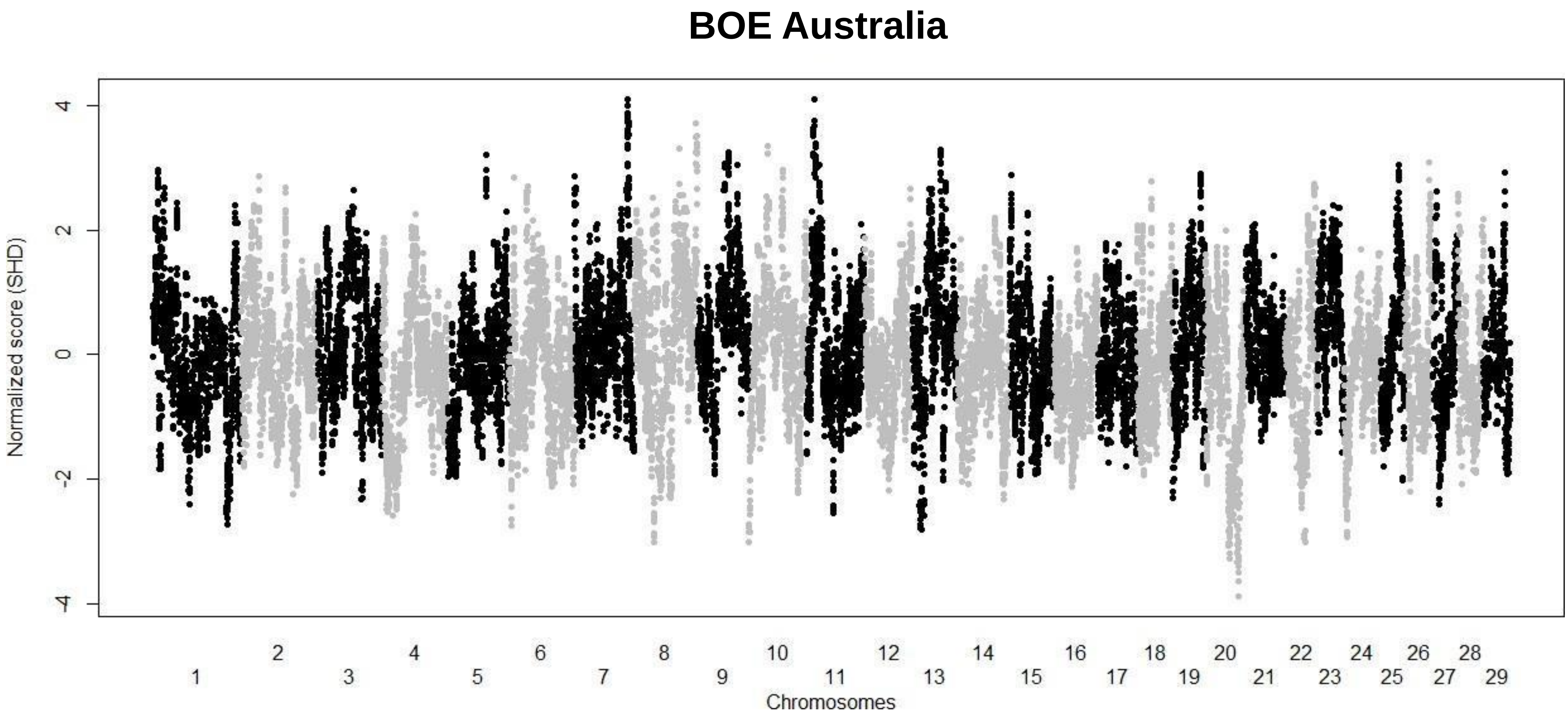
BOE Australia
Normalized score (SHD)
4
2
0
-2
-4
1
2
3
4
5
6
7
8
9
10
11
12
13
14
15
16
17
18
19
20
21
22
23
24
25
26
27
28
29
Chromosomes

BOE Switzerland

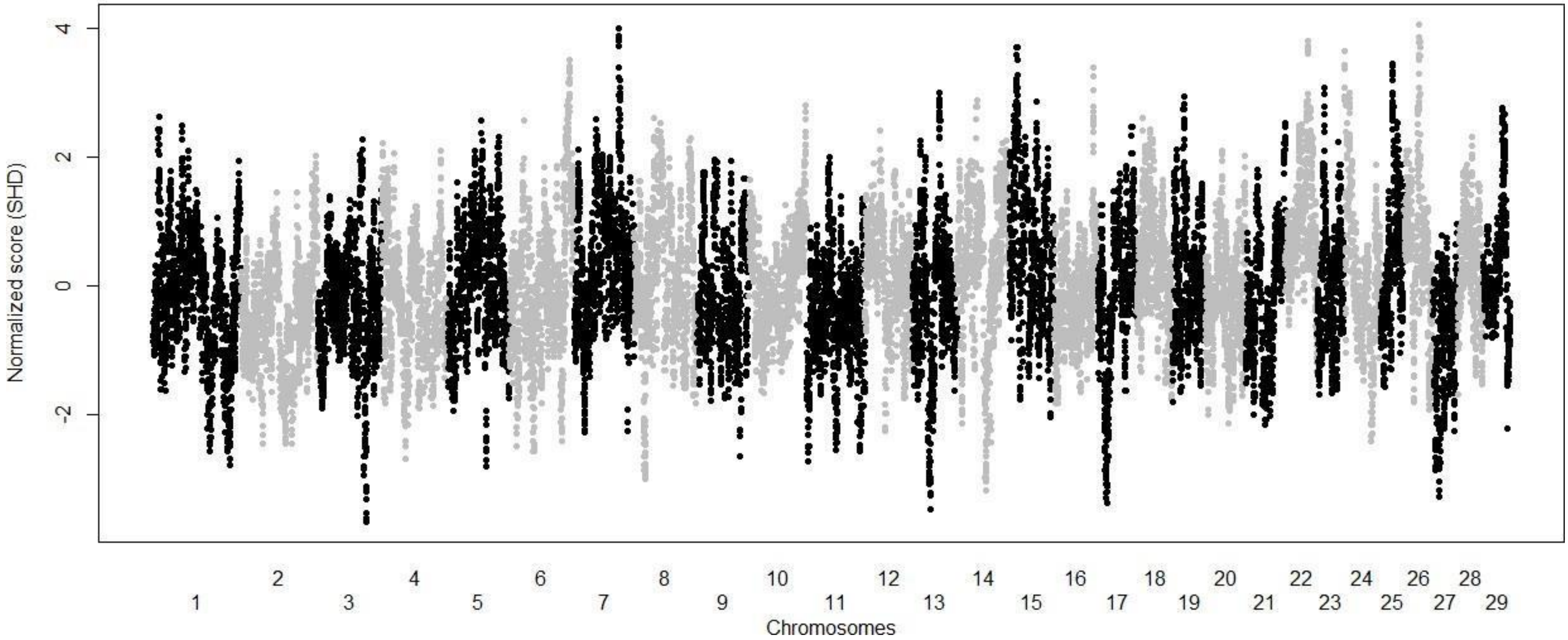

BOE New Zealalnd

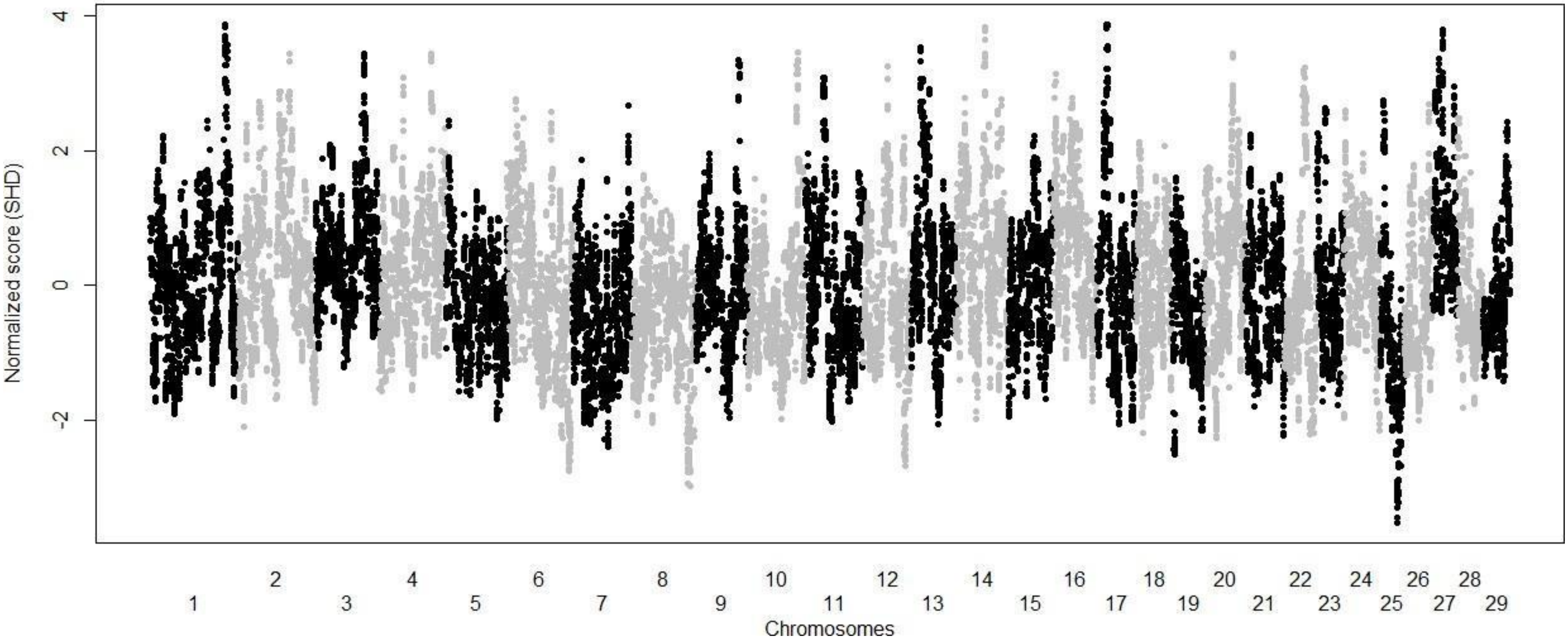

BOE United States

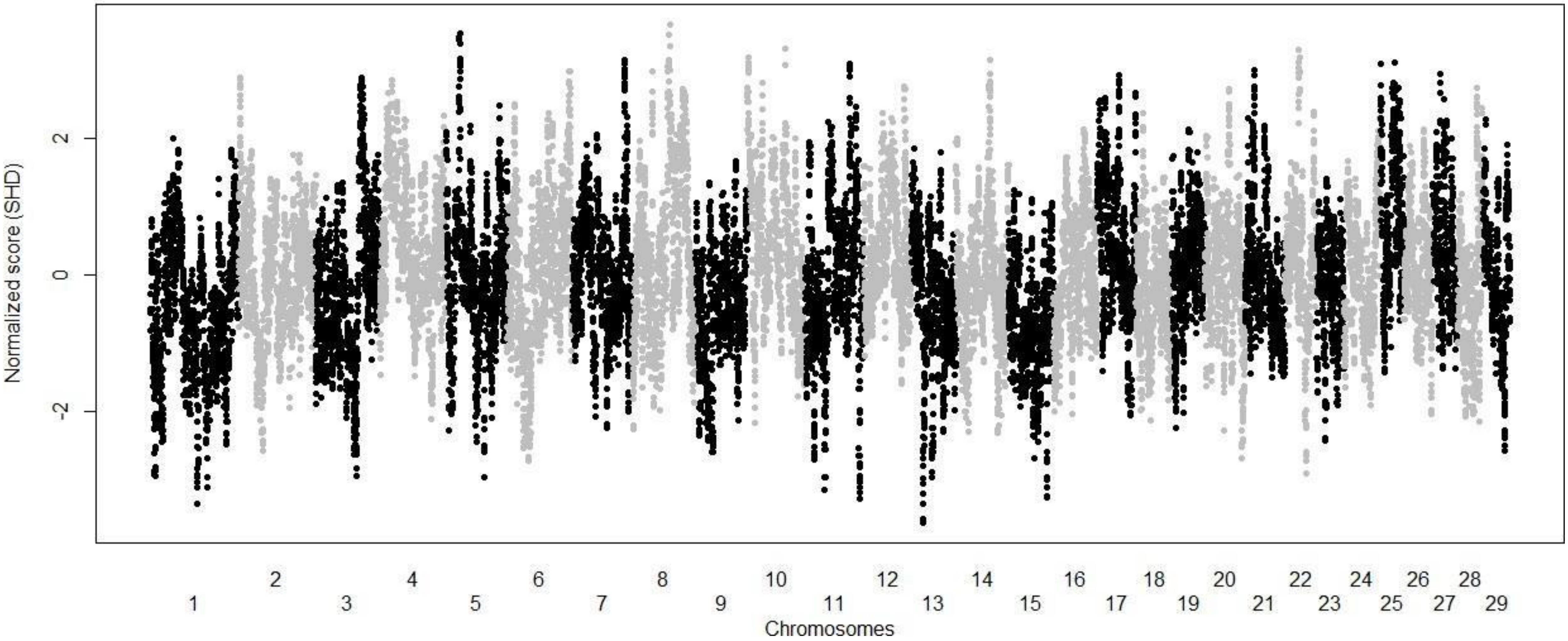

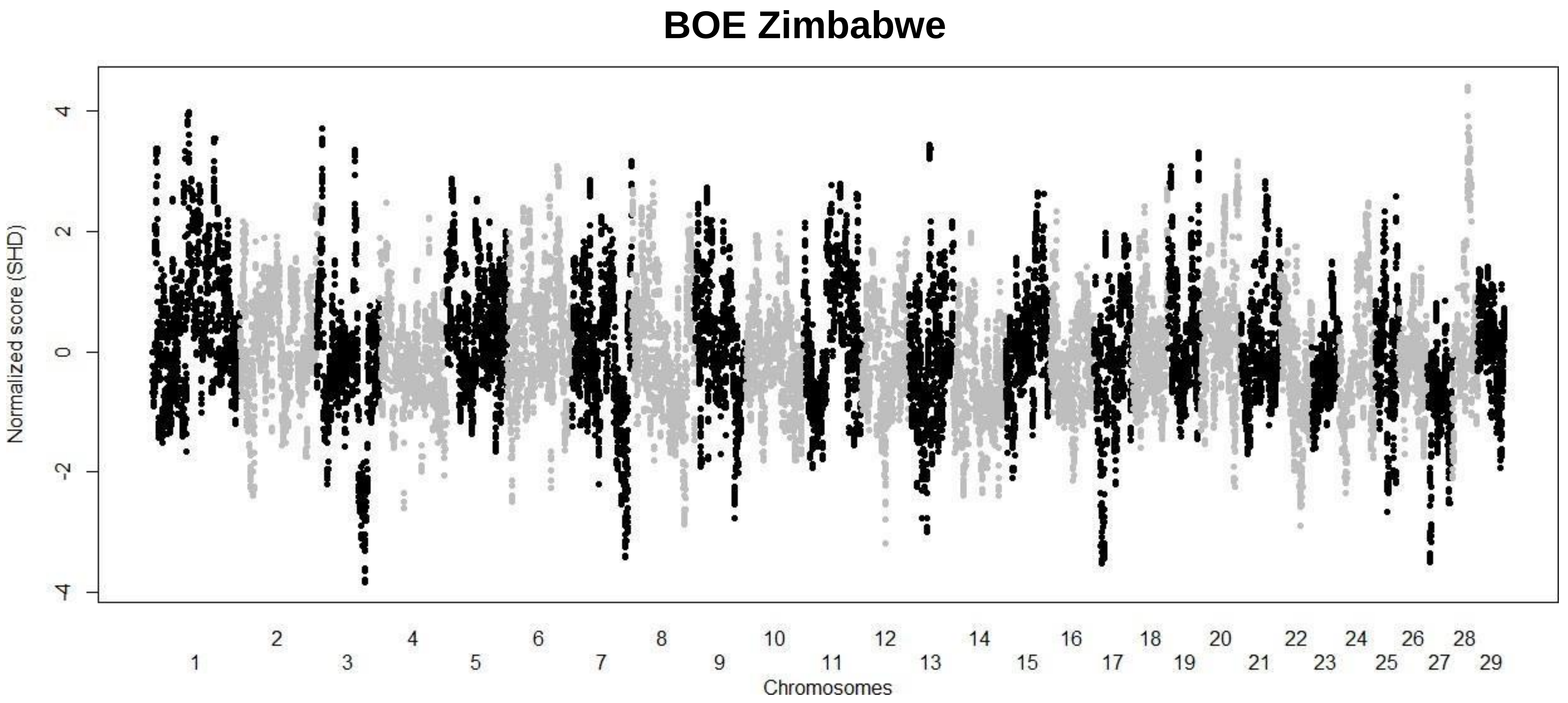
BOE Zimbabwe
Normalized score (SHD)
4
2
0
-2
-4
1 2 3 4 5 6 7 8 9 10 11 12 13 14 15 16 17 18 19 20 21 22 23 24 25 26 27 28 29
Chromosomes

Nubian (NBN)

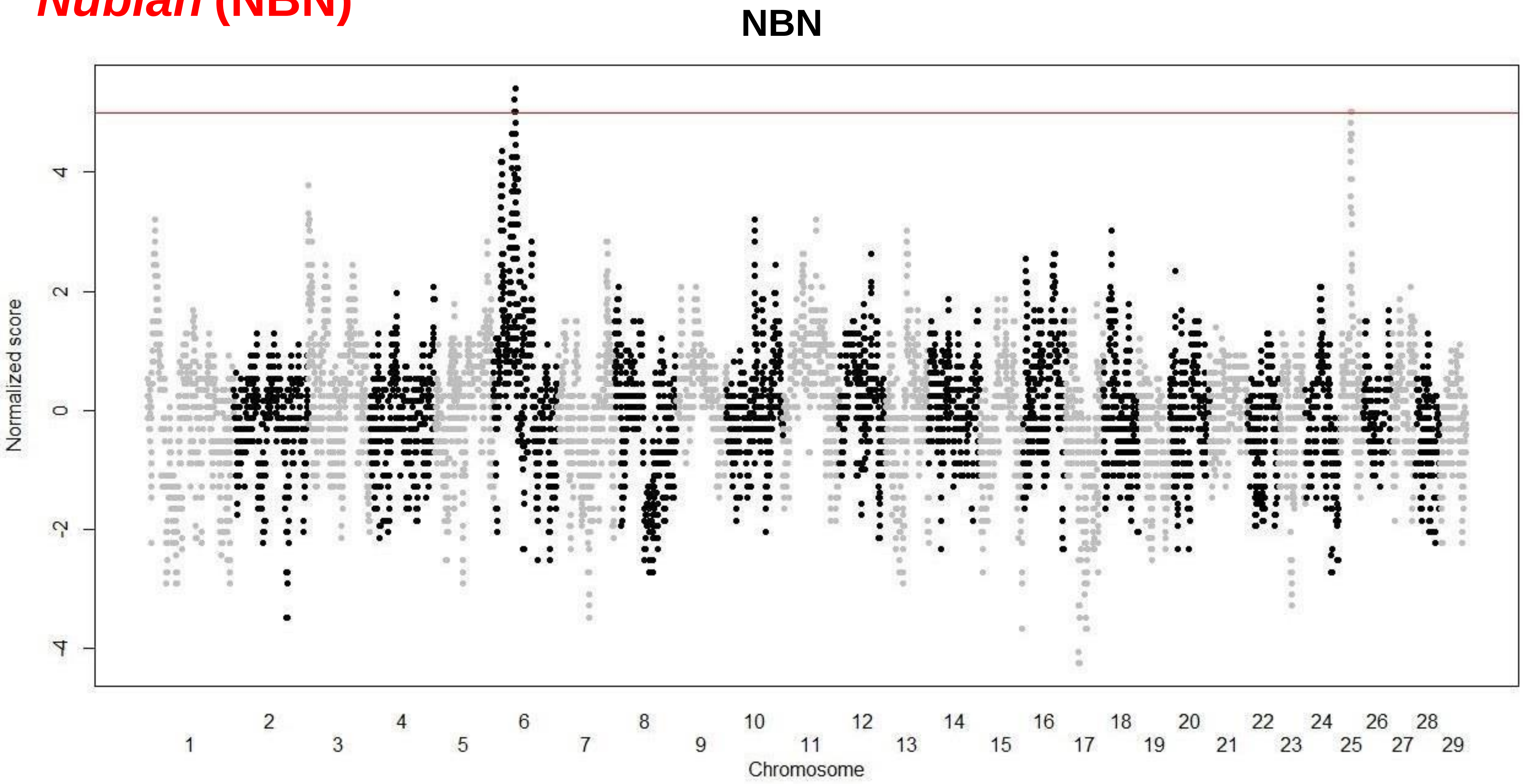

NBN
Normalized score
4
2
0
-2
-4
2
4
6
8
10
12
14
16
18
20
22
24
26
28
1
3
5
7
9
11
13
15
17
19
21
23
25
27
29
Chromosome

Saanen (SAA)

SAA Argentina

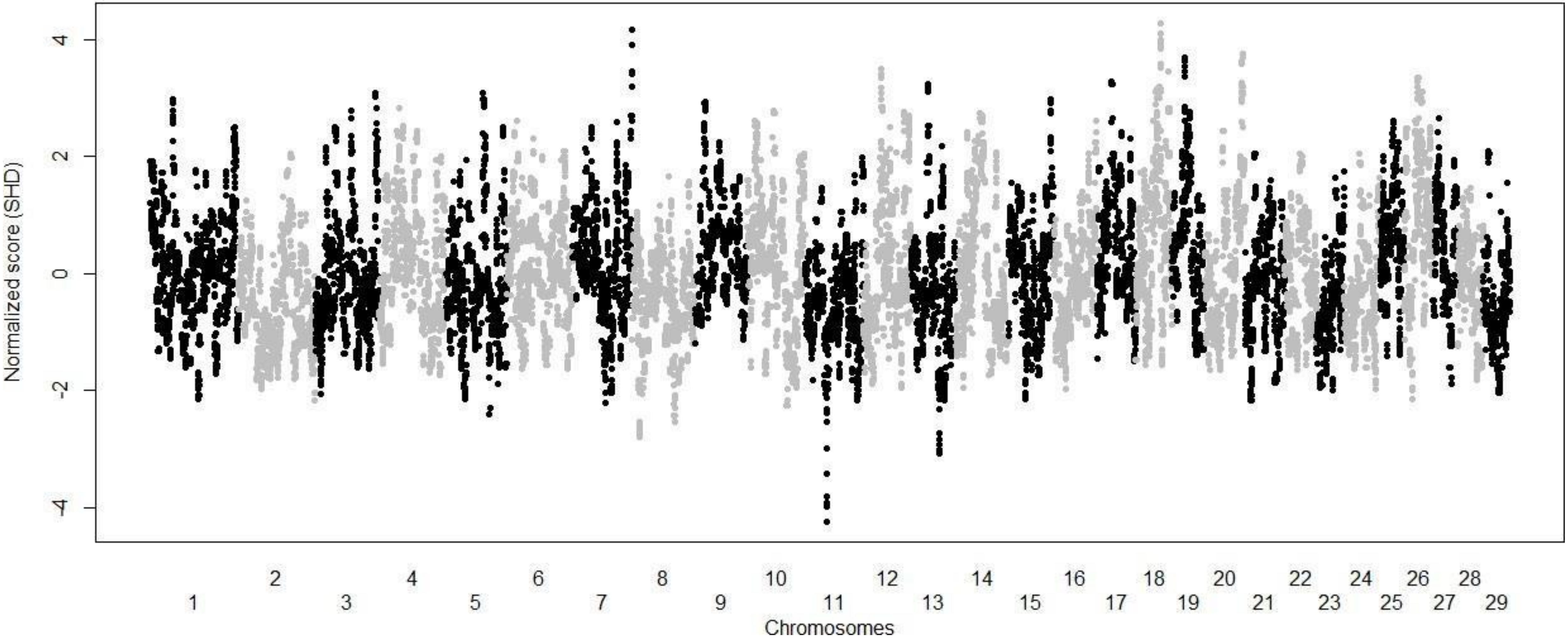

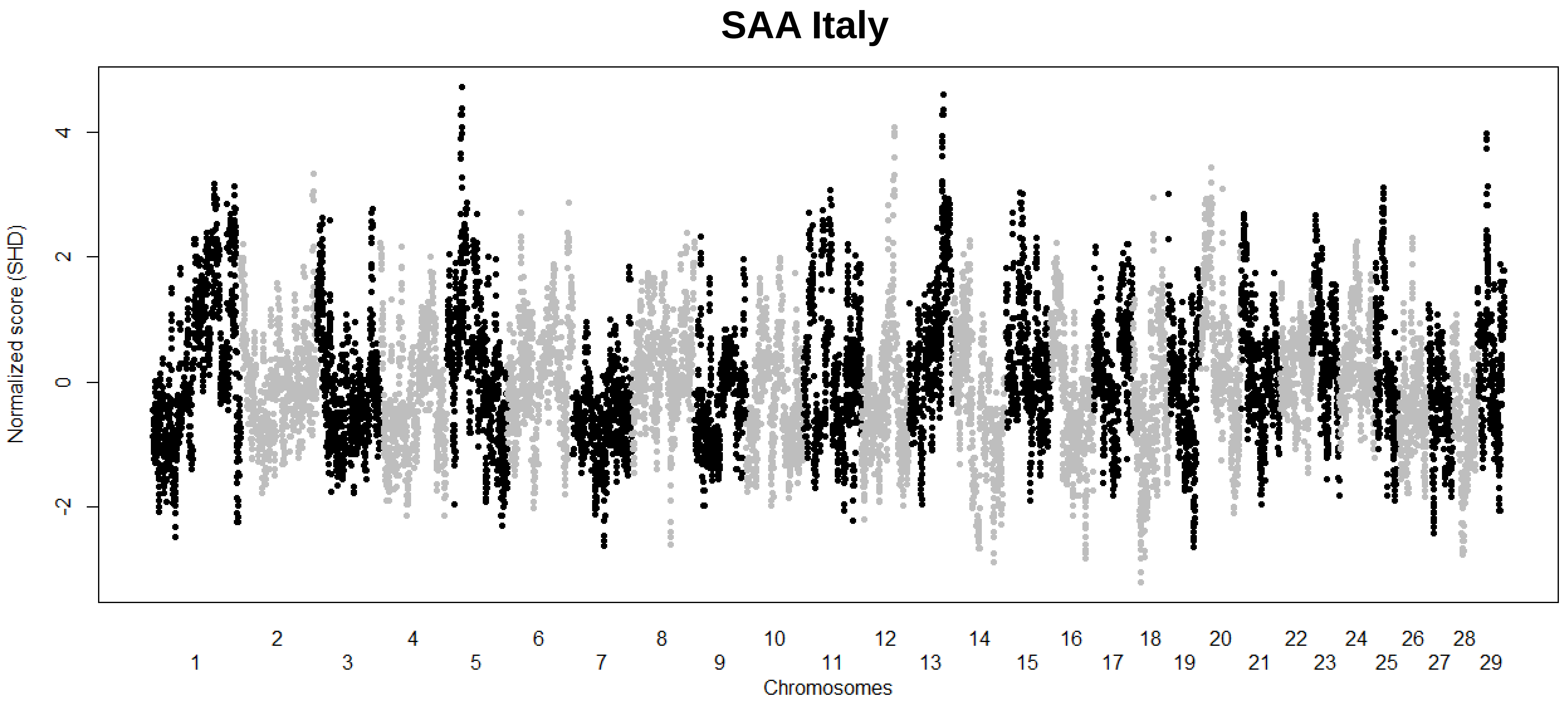
SAA Italy
Normalized score (SHD)
4
2
0
-2
1 2 3 4 5 6 7 8 9 10 11 12 13 14 15 16 17 18 19 20 21 22 23 24 25 26 27 28 29
Chromosomes

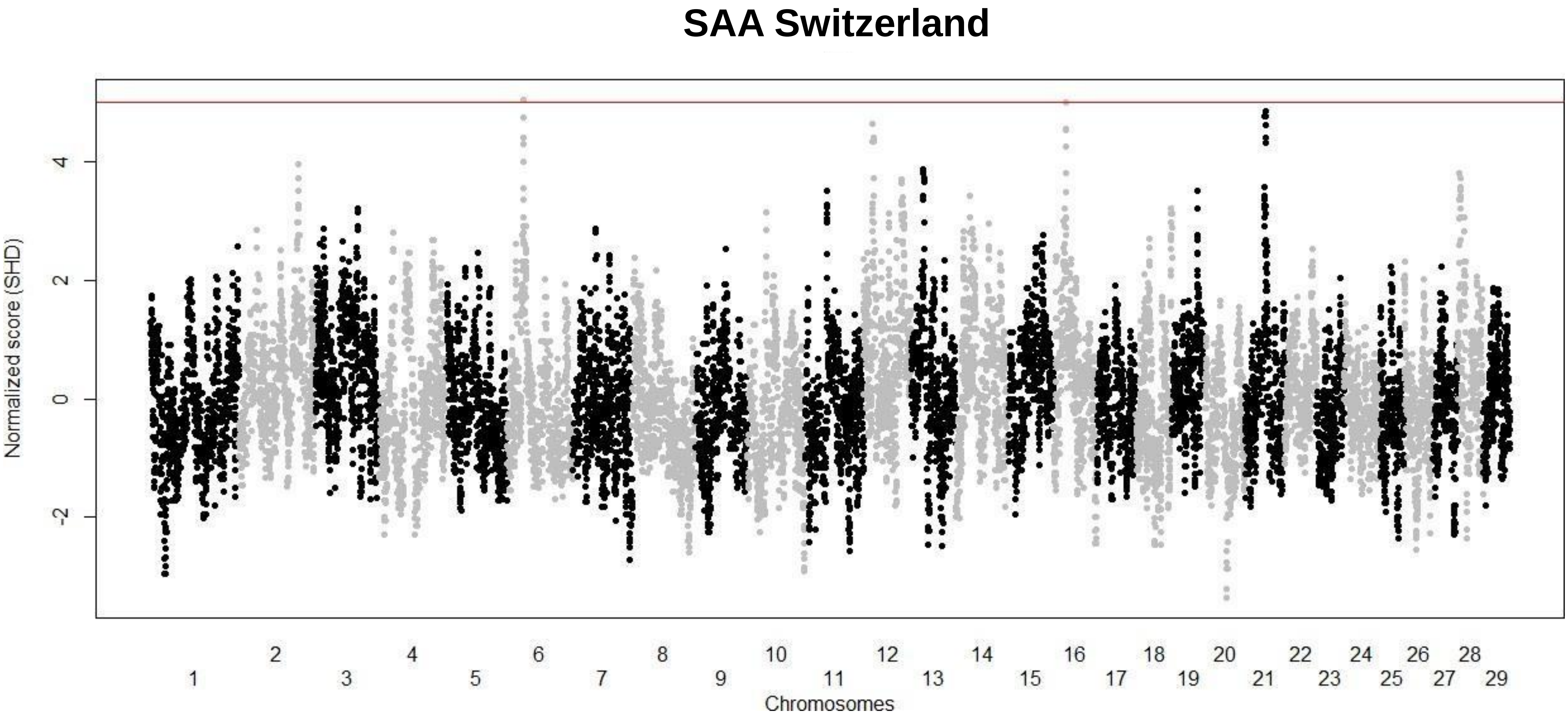
SAA Switzerland
Normalized score (SHD)
4
2
0
-2
1
2
3
4
5
6
7
8
9
10
11
12
13
14
15
16
17
18
19
20
21
22
23
24
25
26
27
28
29
Chromosomes

SAA France

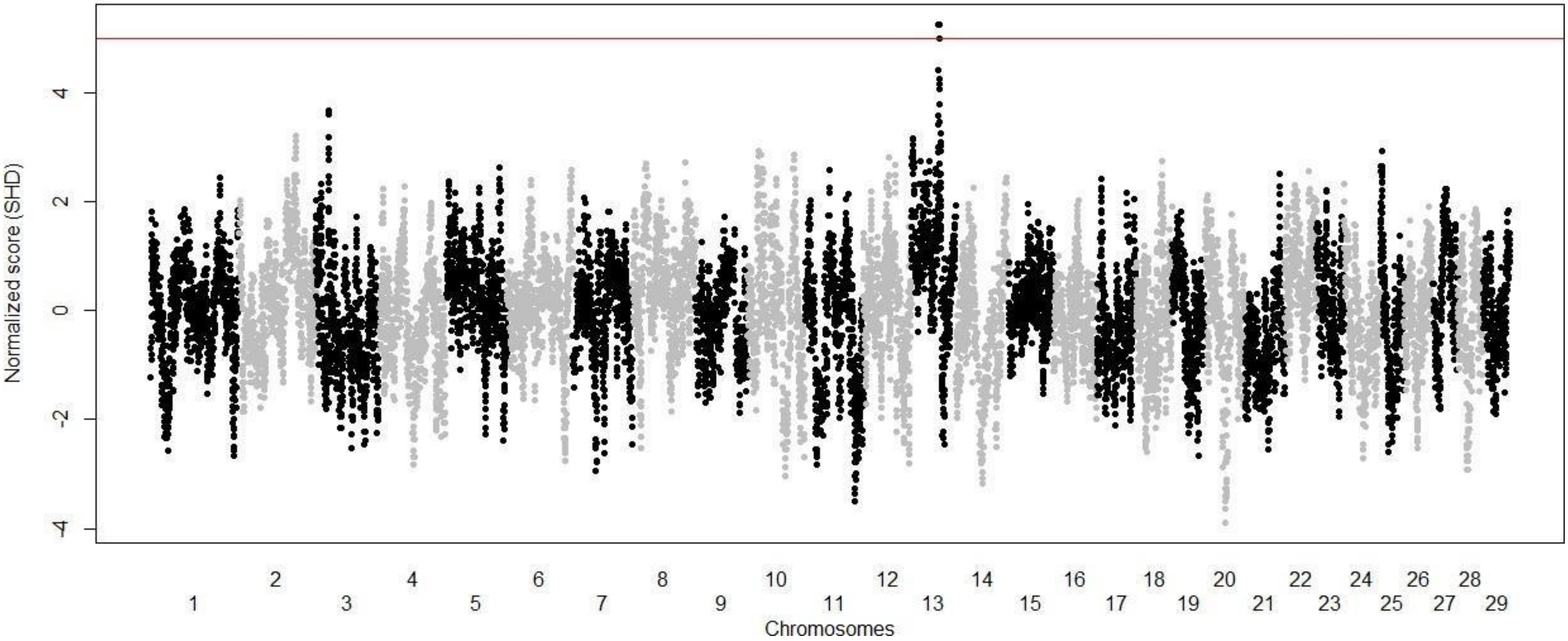
Normalized score (SHD)
4
2
0
-2
-4
1
2
3
4
5
6
7
8
9
10
11
12
13
14
15
16
17
18
19
20
21
22
23
24
25
26
27
28
29
Chromosomes

SAA Tanzania

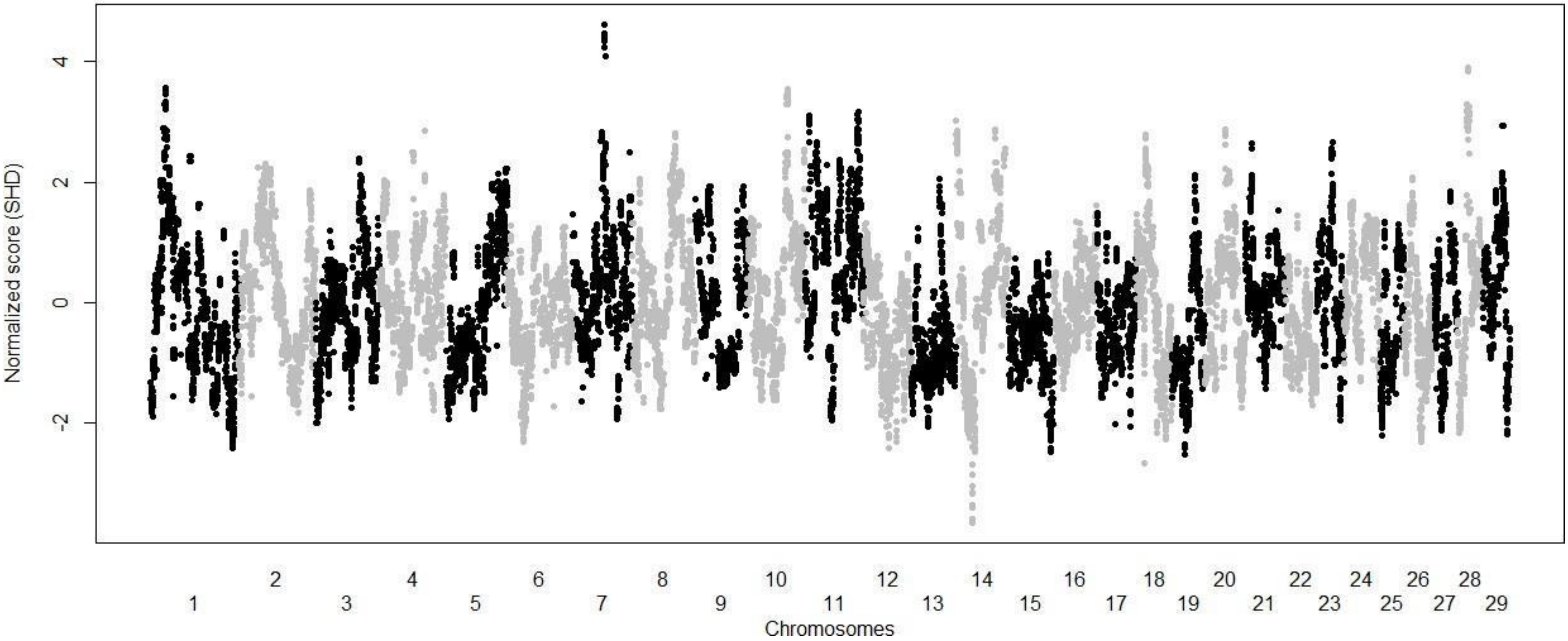

Supplement: Supplementary file 3 — Additional file 3. Comparison of ROH across the breeds raised in different countries. The higher the value on the y axis, the bigger is the difference. The threshold of H = 5 is indicated with a red line. [file 12711_2018_424_MOESM3_ESM.pdf]
